# Connecting Neuronal Cell Protective Pathways and Drug Combinations in a Huntington’s Disease Model through the Application of Quantitative Systems Pharmacology

**DOI:** 10.1038/s41598-017-17378-y

**Published:** 2017-12-19

**Authors:** Fen Pei, Hongchun Li, Mark J. Henderson, Steven A. Titus, Ajit Jadhav, Anton Simeonov, Murat Can Cobanoglu, Seyed H. Mousavi, Tongying Shun, Lee McDermott, Prema Iyer, Michael Fioravanti, Diane Carlisle, Robert M. Friedlander, Ivet Bahar, D. Lansing Taylor, Timothy R. Lezon, Andrew M. Stern, Mark E. Schurdak

**Affiliations:** 1 Department of Computational and Systems Biology, University of Pittsburgh, 3501 Fifth Ave, Suite 3064, Biomedical Science Tower 3, Pittsburgh, PA 15260 USA; 2 National Center for Advancing Translational Sciences, National Institutes of Health, 9800 Medical Center Drive, Rockville, MD 20850 USA; 3 Department of Neurological Surgery, University of Pittsburgh, 200 Lothrop St., UPMC Presbyterian, Suite B-400, Pittsburgh, PA 15261 USA; 4 University of Pittsburgh Drug Discovery Institute, 200 Lothrop St., W950 Biomedical Science Tower Pittsburgh, PA, 15261 USA; 5 Department of Pharmaceutical Sciences, University of Pittsburgh, 3501 Terrace St., Pittsburgh, PA 15261 USA; 6 University of Pittsburgh Brain Institute, 3501 Fifth Ave., 4074 Biomedical Science Tower 3, Pittsburgh, PA 15261 USA

## Abstract

Quantitative Systems Pharmacology (QSP) is a drug discovery approach that integrates computational and experimental methods in an iterative way to gain a comprehensive, unbiased understanding of disease processes to inform effective therapeutic strategies. We report the implementation of QSP to Huntington’s Disease, with the application of a chemogenomics platform to identify strategies to protect neuronal cells from mutant huntingtin induced death. Using the ST*Hdh* ^*Q111*^ cell model, we investigated the protective effects of small molecule probes having diverse canonical modes-of-action to infer pathways of neuronal cell protection connected to drug mechanism. Several mechanistically diverse protective probes were identified, most of which showed less than 50% efficacy. Specific combinations of these probes were synergistic in enhancing efficacy. Computational analysis of these probes revealed a convergence of pathways indicating activation of PKA. Analysis of phospho-PKA levels showed lower cytoplasmic levels in ST*Hdh* ^*Q111*^ cells compared to wild type ST*Hdh* ^*Q7*^ cells, and these levels were increased by several of the protective compounds. Pharmacological inhibition of PKA activity reduced protection supporting the hypothesis that protection may be working, in part, through activation of the PKA network. The systems-level studies described here can be broadly applied to any discovery strategy involving small molecule modulation of disease phenotype.

## Introduction

Huntington’s disease (HD) is a neurodegenerative disease characterized by personality changes, generalized motor dysfunction, and mental deterioration. Symptoms generally develop in the third to fifth decade of life, and the disease ends in dementia and death. HD is rare, affecting 4 to 10 cases in 100,000 people, yet its pathology is strikingly similar to other more common and complex neurodegenerative diseases including Parkinson’s and Alzheimer’s disease. HD displays an autosomal-dominant inheritance and an abnormal extension of the number of glutamine repeats at the N-terminus of a single protein (huntingtin, *HTT*)^1^. Mutant *HTT* (*mHTT*) has been shown to satisfy Koch’s postulates for causing this devastating neurological disorder in which striatal neuronal subtypes exhibit particular but not exclusive vulnerability^1^.

*HTT* (and *mHTT*) is a large protein that interacts with many binding partners^2^, and a number of key pathogenic mechanisms have been described in HD, including aberrant caspase activation, mitochondrial dysfunction^3–7^, ER stress, transcriptional dysregulation, altered calcium signaling, proteasome inhibition, defects in vesicle transport, and altered neurotransmitter release and activity^1,3,4^. However, despite knowledge of the causal gene, and the existence of multiple rodent models that recapitulate key molecular, cellular, and behavioral phenotypes of the human disease^1^, drug-like molecules that can reduce *mHTT* protein expression, increase its clearance, or prevent mutant *HTT*-induced cell death have yet to be successfully identified in clinical trials. The slow progress toward effective therapy has been attributed to an insufficient knowledge of those biological functions of the *mHTT* protein that are critical in HD. Furthermore, resulting pleiotropic effects have made it difficult to distinguish whether particular aspects of *mHTT*-associated dysregulation are actually mechanistically linked to disease progression (i.e., pathogenic), epiphenomena, or disease-ameliorating compensatory effects.

**Figure 1:**
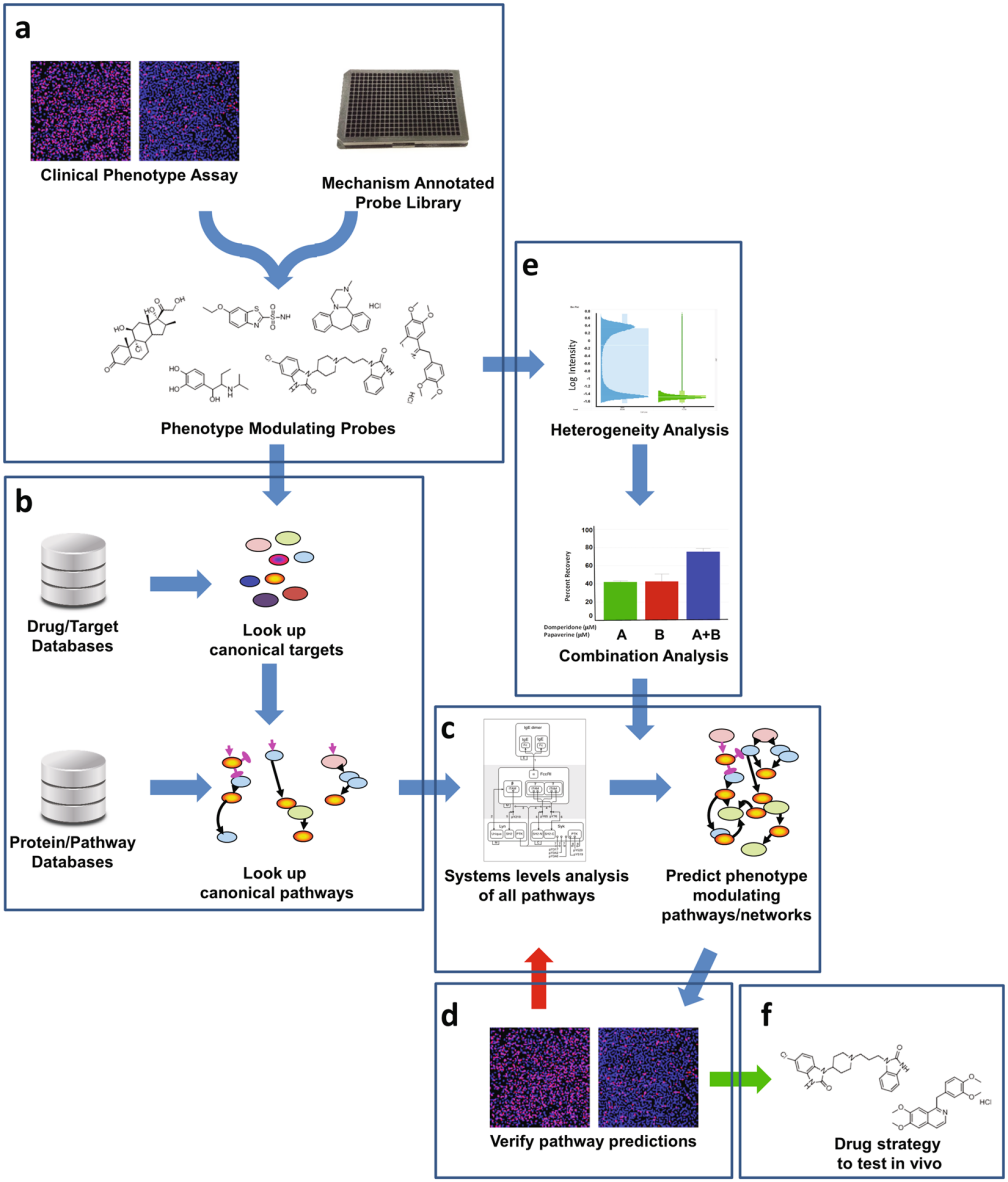
Chemogenomics component of the QSP platform. (**a**) Libraries of mechanism annotated probe compounds are screened in a clinically relevant phenotypic assay to identify phenotype modulating probes. (**b**) Targets for the active probes are identified from various drug-target databases and then are associated with biological pathways using information from protein-pathway databases. (**c**) Using a systems level analysis of all pathways identified, computational analysis is performed to predict the optimal modulating pathways/networks based on the activity of the respective probes (i.e., activation or inhibition of pathways in relation to the known effects of the pathway on the phenotype). (**d**) Predicted pathway/network hypotheses are tested in phenotypic assays by i) testing additional compounds known to modulate the pathways, ii) testing compounds predicted by advanced machine learning methods that will modulated the pathway, iii) modulate pathways by knock-down and knock-in approaches, and/or iv) evaluate probes in pathway specific phenotypic assays. If pathways are not confirmed, then the hypothesis is refined with the new information gained from the testing, additional probes are identified, and the new hypothesis is tested. If pathways are confirmed, then the active probes are advanced to *in vivo* testing. (**e**) At the initial screening analysis stage, the heterogeneity of phenotype modulating response is assessed. If no heterogeneity is detected, then proceed as above. However, if heterogeneity is detected, then hypotheses are developed and tested to characterize the basis of the heterogeneity (e.g., effects of combinations of different compounds). The information gained from the heterogeneity analysis is used to inform the prediction of the phenotype modulating pathways/networks. (**f**) The outputs of this strategy are i) a systems level understanding of the pathways/networks involved in the clinically relevant phenotype which enables the design of optimal therapeutic strategies, and ii) probes/drugs that can be advanced to *in vivo* and clinical testing.

Treating HD, or any complex disease, requires a thorough understanding of its mechanisms of progression. Identifying disease mechanisms is hindered by epistasis, pleiotropy and heterogeneity^8^, all of which are intrinsic and often confounding characteristics in complex diseases^9^. An attractive path to systematically understanding mechanisms of disease progression is Quantitative Systems Pharmacology (QSP), an approach that integrates and iterates computational and experimental methods to determine molecular pathogenesis^10,11^. A chemogenomics component of QSP involves perturbing disease phenotypes in clinically relevant assays with mechanistically annotated compounds, and using the known mode-of-action of active compounds to infer cellular pathways that are related to the disease and its modulation (see Fig. 1). Concordance in the perturbation of a disease phenotype among a set of structurally diverse chemical probes sharing an annotated common mechanism can provide compelling evidence for the role of a particular target/pathway in the molecular etiology^12^. In turn, a discordance with such a probe set could lead to the identification of a novel disease-specific mechanism. This finely tunable pharmacological approach is complementary to genetic approaches^12^.

We initiated the QSP approach and implemented the chemogenomic strategy investigating the protective effects of small molecule probes with diverse canonical molecular mechanisms of action in a well-established striatal neuronal cell model (ST*Hdh* ^*Q111*^) for HD^13^. The objective of this work is to generate testable hypotheses regarding disease mechanism and potential mechanisms involved in protection of neuronal cells from mHTT dependent toxicity. We report here on the first two iterations of the QSP approach. We identified a number of small molecule probes with a range of distinct canonical mechanisms that protect the ST*Hdh* ^*Q111*^ cells from *mHTT*-induced death. We found that the response of the cell population to most of the compounds was heterogeneous, i.e., not all of the cells within a population were protected by the compounds, which was not unexpected since heterogeneous responses to compounds are common^14^. Interestingly, testing of combinations of moderately active compounds identified specific combinations that synergistically increased the efficacy of protection. Analysis of the canonical mechanisms of 10 compound pairs that synergistically protected ST*Hdh* ^*Q111*^ cells showed a convergence of pathways leading to the activation of PKA and PKG. Cytoplasmic phospho-PKA levels were lower in ST*Hdh* ^*Q111*^ than in the wild type ST*Hdh* ^*Q7*^ cells under stress conditions, and these levels were increased by several of the protective compounds. In addition, co-incubation with the PKA inhibitor H89 inhibited the protective effects of the compounds. Our results suggest that active PKA may have a role in the protective effects of these compounds. The information gained from the annotated compounds and combination analysis provided input for inference of neuronal cell protective pathways.

## Results

### Characterization of neuronal cell protective compounds in the ST*Hdh*^*Q111*^ model

**Figure 2:**
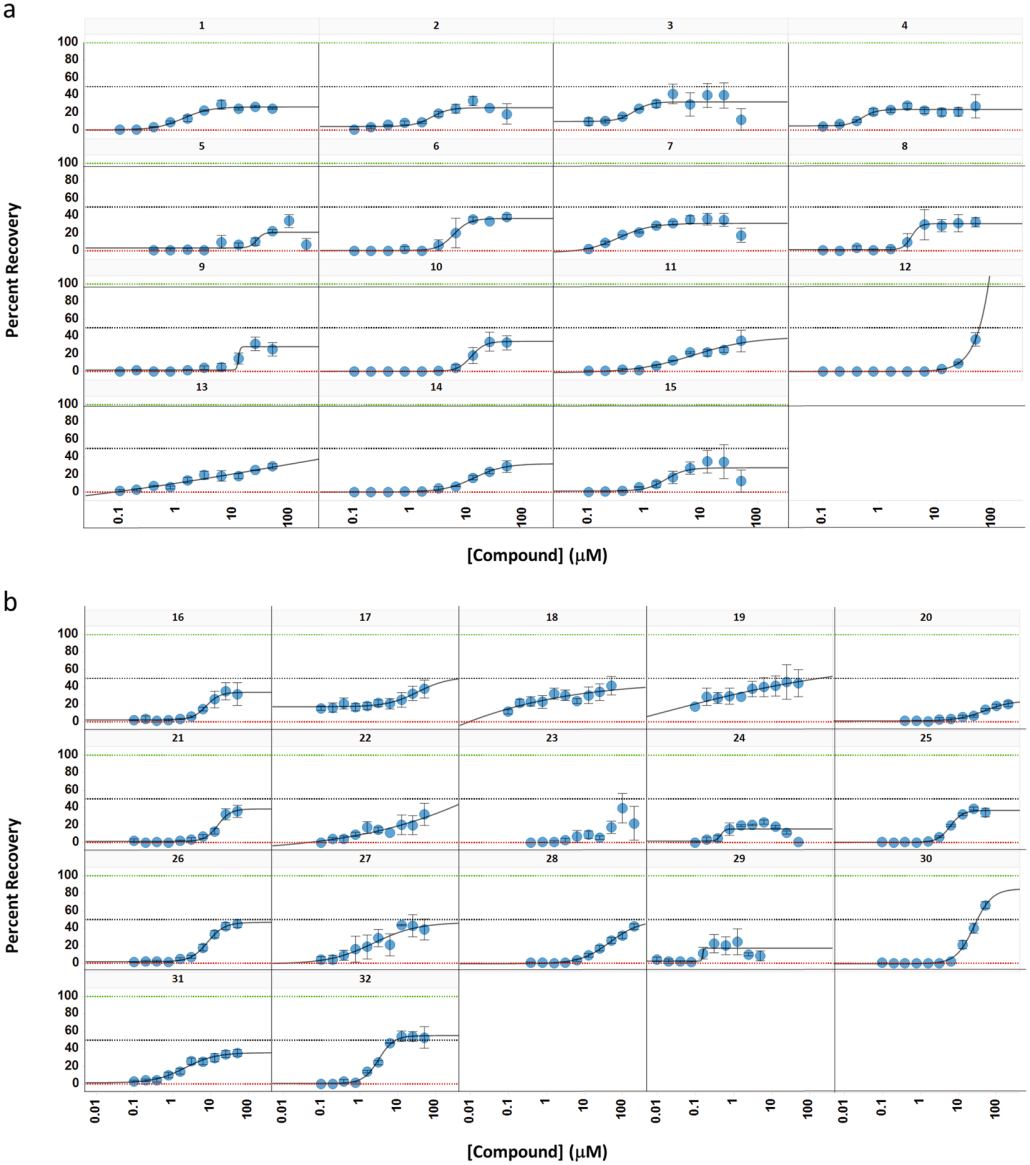
Compounds with confirmed neuroprotective activity in the ST*Hdh* ^*Q111*^ model. Compound titrations were tested for protective activity in the 384-well PI assay. Compounds representing a diverse set of canonical mechanisms show only partial efficacy in protecting ST*Hdh* ^*Q111*^ cells from *mHTT* induced cell death. (**a**) Compounds reported in the literature to be associated with central nervous system (CNS) activity: 1) 3-tropanyl-indole-3-carboxylate hydrochloride; 2) Benztropine mesylate; 3) Cyproheptadine hydrochloride; 4) Domperidone; 5) Isoetarine mesylate; 6) JWH-015; 7) Loxapine succinate; 8) Meclizine; 9) Mianserin hydrochloride; 10) PD 168,077 maleate; 11) Quipazine, N-methyl-,dimaleate; 12) Ruthenium red; 13) SB 203186; 14) Triprolidine hydrochloride; 15) Vinpocetine. (**b**) Compounds reported to be associated with non-CNS activity: 16) (Z)-Gugglesterone; 17) Beclomethasone; 18) Betamethasone; 19) Budesonide; 20) Ethoxzolamide; 21) Flutamide; 22) Hydrocortisone; 23) Lansoprazole; 24) Lonidamine; 25) m-Iodobenzylguanidine hemisulfate; 26) Papaverine hydrochloride; 27) Prednisolone; 28) Sodium Nitroprusside; 29) Vorinostat; 30) Tetradecylthioacetic acid; 31) Triamcinolone; 32) U-83836 dihydrochloride. Results are from triplicate samples run in at least two independent experiments (Error bars are +/−SE).

We employed the well-established ST*Hdh* ^*Q111*^ cell model for HD^13,15^ to identify compounds that would protect neuronal cells from *mHTT*-dependent cell death. In this model, serum deprivation (which mimics the clinical stress of growth factor deprivation) of the ST*Hdh* ^*Q111*^ cells containing *mHTT* results in cell death, whereas under the same conditions the ST*Hdh* ^*Q7*^ wild type cells are resistant to cell death. The propidium iodide (PI) readout enables an unbiased assessment of cell death by measuring an irreversible step that is common to all cytotoxic mechanisms^16^. Under serum-depleted conditions, ~50 percent of the ST*Hdh* ^*Q111*^ cells underwent cell death as evident by positive nuclear PI staining, compared to less than 10 percent of the wild type ST*Hdh* ^*Q7*^ cells (Supplementary Figure S1). From screens of the LOPAC^1280^ library, the NCATS Pharmaceutical Collection^17^, and a library of 83 compounds computationally predicted to be neuroprotective (see Methods), we confirmed the activity of 32 compounds (Fig. 2).

Interestingly, the level of protection afforded by the majority of the compounds did not reach 100%, exhibiting plateaus in the dose response curves between 30% and 50%. We verified that the neuronal cell protection observed was not an overestimate simply due to an undetectable loss of dead cells (Supplementary Figure S2), and that partial protection was not simply due to limited solubility within the efficacious dose range (Supplementary Table S1). The spectral properties of PI are red shifted relative to the majority of small molecule compounds, thus avoiding compound interference (quenching). Preliminary analysis of the hit compounds in an LDH-based cell death assay with a format and readout distinct from that of PI showed similar curves for the hit compounds (data not shown) as seen in the PI assay. For a subset of compounds, we also examined the direct effect on quenching the PI signal and found that quenching did not occur (Supplemental Figure S3). These results indicate that the partial protection was an outcome of compound perturbation of *mHTT*-induced biology under these experimental conditions.

We searched the DrugBank and STITCH databases for the canonical targets of the 32 active compounds. Ten compounds had no known targets in either database; the remaining set of 22 displayed a diverse range of canonical mechanisms of action targeting 75 proteins on a number of pathways (Supplemental Tables S2 and S3). Many of the canonical targets have known functions that are critical to CNS activity. For example, histamine receptors, the target of 7 hit compounds, are associated with multiple neuropsychiatric disorders. Receptors of the neurotransmitters serotonin and dopamine are also targets of several of our hit compounds. Nine active compounds did not share any targets with other hits in the screen, suggesting that either multiple mechanisms are capable of conferring neuronal cell protection or some of the active compounds operate through shared non-canonical mechanisms.

### Combinations show enhanced protective effects

**Figure 3:**
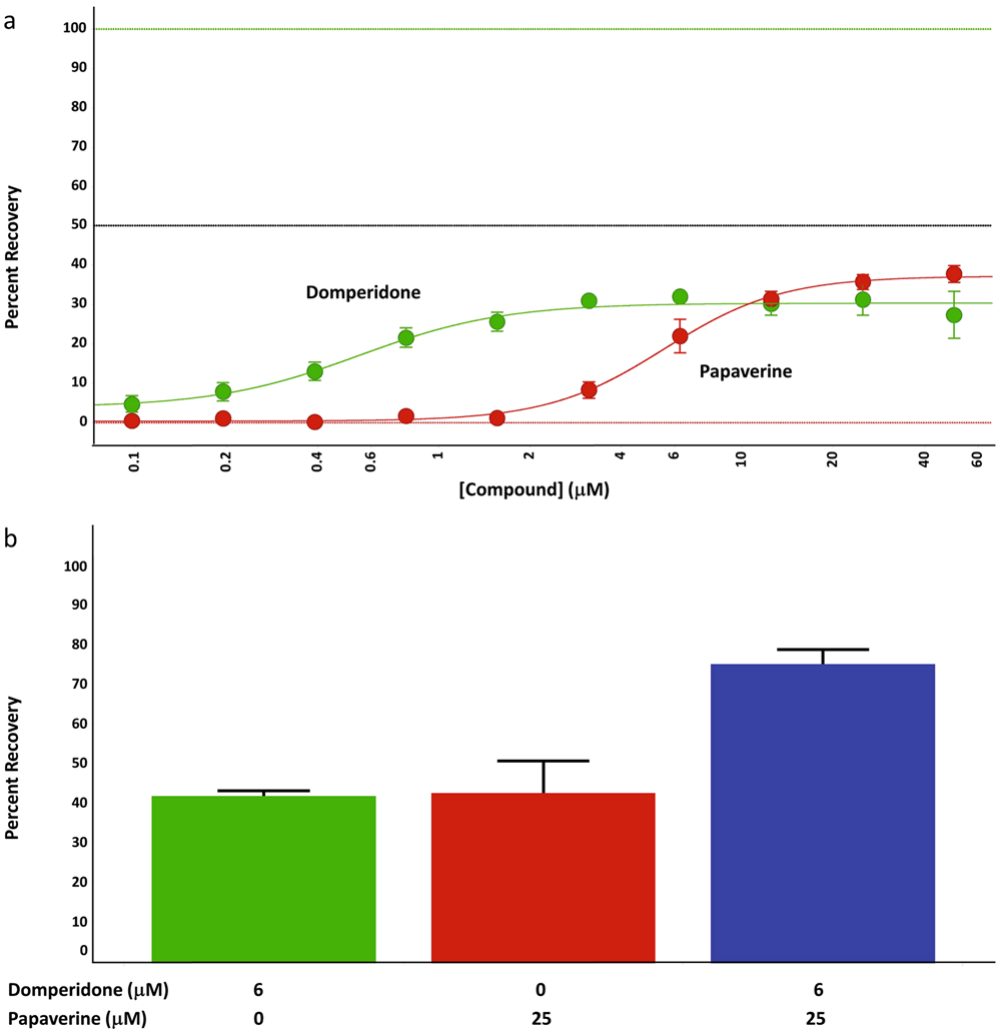
Combinations of probes with different canonical mechanisms provide enhanced protection of ST*Hdh* ^*Q111*^ cells. (**a**) Using domperidone and papaverine as an example, concentrations of compounds that were on the plateau of the activity curve were chosen for combination experiments. In this example, 6 μM domperidone and 25 μM papaverine were selected. (**b**) Compounds were combined and tested in the 384-well PI assay. The percent activity of the combination was compared with the activity of the single compounds run in parallel, and the ratio of the combined activity to that of the single compound with the highest activity is taken as the combination ratio. For domperidone and papaverine the combination ratio shown here is 1.74 (n = 3 independent experiments, error bars are +/−SE). The combination experiments in panel  b were run independently from the titration experiments in panel a.

**Figure 4:**
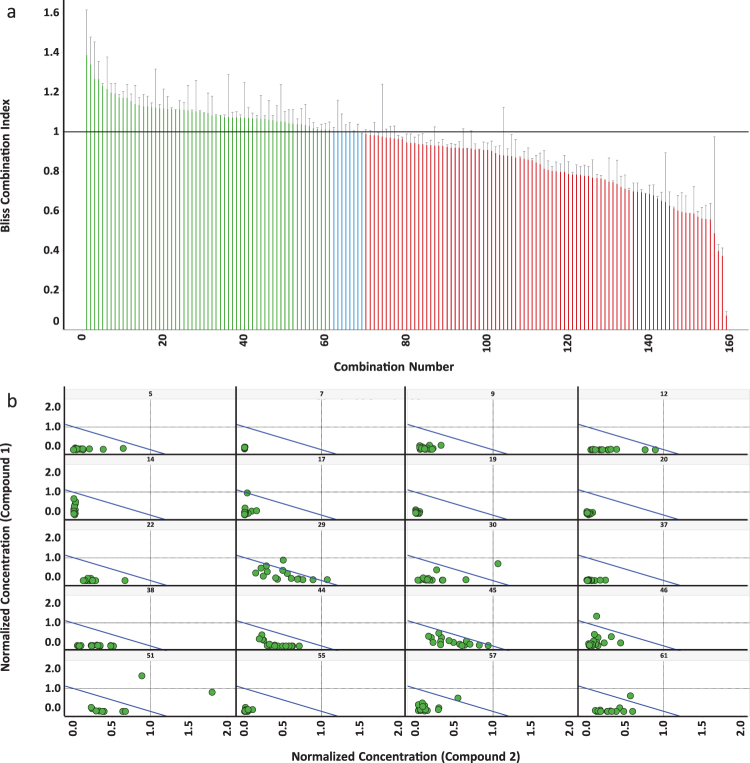
Combinations of probes show synergistic protection in ST*Hdh* ^*Q111*^ cells. (**a**) Active LOPAC probes were screened in combinations using a single concentration of each probe. Combination numbers refer to the combinations listed in Supplementary Table S4. Bliss Independence Model analysis indicated 61 combinations to be synergistic in the single concentration combination screen. The Bliss Independence Model compares the predicted activity of probe combinations to the experimentally observed activity of the combination^51^. The Bliss Combination Index (BCI) is the ratio of the observed combination activity to the predicted combination activity based on the activity of the individual compounds. A BCI > 1 indicates synergy (green bars) and a BCI < 1 indicates antagonism (red bars), while BCI = 1 indicates additivity (blue bars). To accommodate additive BCI calculations not equaling 1 exactly, a cutoff of 0.99–1.01 was assigned to classify synergy and antagonism. (Results from at least 2 independent runs, error bars are the Median Absolute Deviation). (**b**) 20 probe pairs were selected and tested using 4 different concentrations, 2 each from the plateau and linear portions of the single compound concentrations curves. Curves were analyzed by the method of Chou and Talely^20^, and the isobolograms are plotted. Points below the diagonal line represent synergistic activity of the two compounds (n = 2 independent runs). The panel numbers are the Combination Numbers for the combinations tested listed in Supplementary Table S4.

The diversity of canonical mechanisms of the compounds exhibiting protection and the partial maximal protection for any one compound suggested the presence of more than one protective mechanism, where the sufficiency for any one mechanism to afford complete protection in an individual cell varied across the cell population. To explore this further, we asked if the efficacy of neuronal cell protection could be enhanced with pairwise combinations of compounds with different canonical mechanisms. We implemented the combination screen using 25 of the confirmed LOPAC hits and ethoxzolamide, one of the computationally predicted hits. We screened 268 compound pairs with each compound at a single concentration that was on or near the plateau of the activity of the respective individual compound, and compared the percent recovery (i.e., protection from cell death) of compound combinations to that of the individual compounds (See Fig. 3 as an example). From the 268 pairs tested, 109 pairs showed enhanced toxicity. Toxicity is defined as the loss of cells from the well using the criteria of total cell number being below 3 SD of the total number of cells in the DMSO controls. For the remaining 159 pairs of combinations (Supplementary Table S4), we determined if the combination effect was additive, synergistic, or antagonistic by calculating a combination index using the Bliss Independence Model^18,19^. We found that 61 combination pairs in this screen had synergistic interactions (Fig. 4a, Supplementary Table S5) while 90 pairs were calculated to be antagonistic and 8 appeared to be additive. We verified the synergistic assessment of the single point analysis by selecting 20 pairs of compounds, testing them in concentration response experiments, and calculating the combination index using the method of Chou and Talalay^20^. All of the pairs tested in this analysis were determined to be synergistic (Fig. 4b). This test gave us confidence in the assessment of the other combinations used in the single point experiments.

Bliss independence (additivity) exists when the effects of compounds are statistically independent: applying one compound neither enhances nor diminishes the effects of the other. Whereas independence implies completely separate mechanisms, synergism and antagonism each imply a relationship between mechanisms, either within cells, across the population, or both. Antagonism at the population level can occur between compounds that share a therapeutic target and therefore compete with each other. Similarly, synergy can arise from mutually exclusive mechanisms manifested in non-overlapping cell subpopulations. Any given cell will respond to only one compound in the synergistic pair, minimizing the number of cells that are redundantly protected by both compounds. The results of our combination screens support these mechanisms. Forty-five of the 90 antagonistic pairs of compounds identified in our screen have known targets. Fifteen of these pairs (33%) are compounds that share at least one target. In contrast, target sharing is observed in only 2 of the 41 synergistic pairs (5%) with known targets.

### Inferring protection-relevant pathways from the compounds’ canonical mechanisms

The mechanistic diversity and synergistic effects of the compounds affording protection from *mHTT*-induced cell death suggested functional interrelationships among their targets. Synergy can arise from mechanistic interactions within the cell if two compounds affect distinct upstream effectors of a common mechanism. Each provides partial protection to the cell, and both, when combined, may confer sufficient protection to permit survival. Alternatively, targets on the same pathway may be heterogeneously expressed in a correlated fashion within the population, causing some cells to modulate the targeted pathway in response to one compound, and other cells to modulate the same pathway through an alternative mechanism. Assays with binary readouts, such as the PI assay used here, mask the mechanistic origins of synergy. We therefore turn to pathway analysis to investigate whether the observed synergy results from pathway convergence within cells, or from mutually exclusive modulation of pathways across a heterogeneous population.

**Figure 5:**
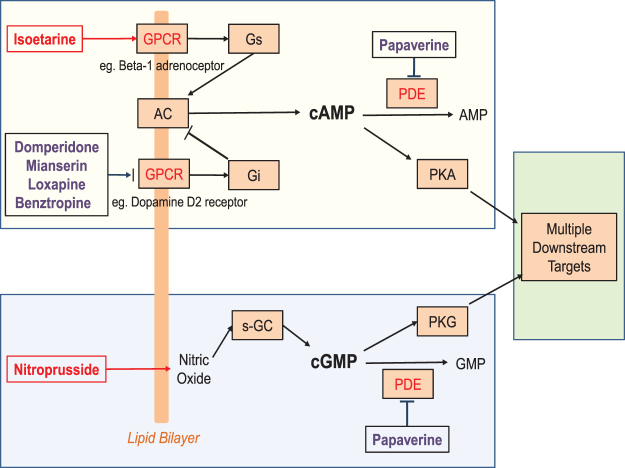
Neuroprotective pathway hypothesized using the canonical targets of compounds that showed synergistic activity (see text for description).

Sixteen compounds were associated with the 41 synergistic pairs that had known targets. In 21 of these synergistic pairs, the compound targets shared at least one pathway as annotated in the KEGG database. The canonical targets for compounds in 10 of these 21 pairs converged on either the cAMP/PKA signaling pathway, the cGMP/PKG signaling pathway, or both (Fig. 5 and Supplementary Table S6). At random, we would expect to find only three synergistic pairs on these pathways (enrichment factor of 3.73, see Methods). No other pathway contained targets of more than four synergistic pairs, as was seen in both calcium signaling and Gap junction pathways.

We hypothesized that synergistic neuronal cell protection could arise in pairs of compounds that had the same effect on cAMP or cGMP signaling, but through distinct complementary mechanisms. For example, isoetarine is an agonist of the β_1_ adrenergic receptor (β_1_AR)^21^, which couples to Gs and stimulates conversion of ATP to cAMP by adenylate cyclase (AC). Benztropine is an antagonist of the M1 muscarinic receptor^22^, blocking the Gi-coupled inhibition of AC activity. Thus, both compounds have the potential to increase PKA activity, but through different mechanisms: isoetarine stimulates AC, and benztropine antagonizes an AC inhibitor. Another example is the synergistic combination of domperidone and papaverine. Similar to benztropine, domperidone can elevate cAMP levels by antagonizing D2R^23^. Papaverine inhibits the phosphodiesterases PDE4B and 10A^24^, reducing the hydrolysis of cAMP into AMP. The net effect of this combination is to increase cAMP levels and PKA activity through two complementary mechanisms. Thus, increasing cAMP levels and correspondingly activated PKA levels or by analogy cGMP/PKG levels may lead to cytoprotection. Multiple compounds targeting the same pathway is distinct from multiple compounds interacting with the same target. Whereas in the latter compounds may compete for the same target site and thus do not lead to enhanced modulation of the target, modulating different points on a pathway can result in synergy enabling more control in regulating the output of the pathway.

**Figure 6:**
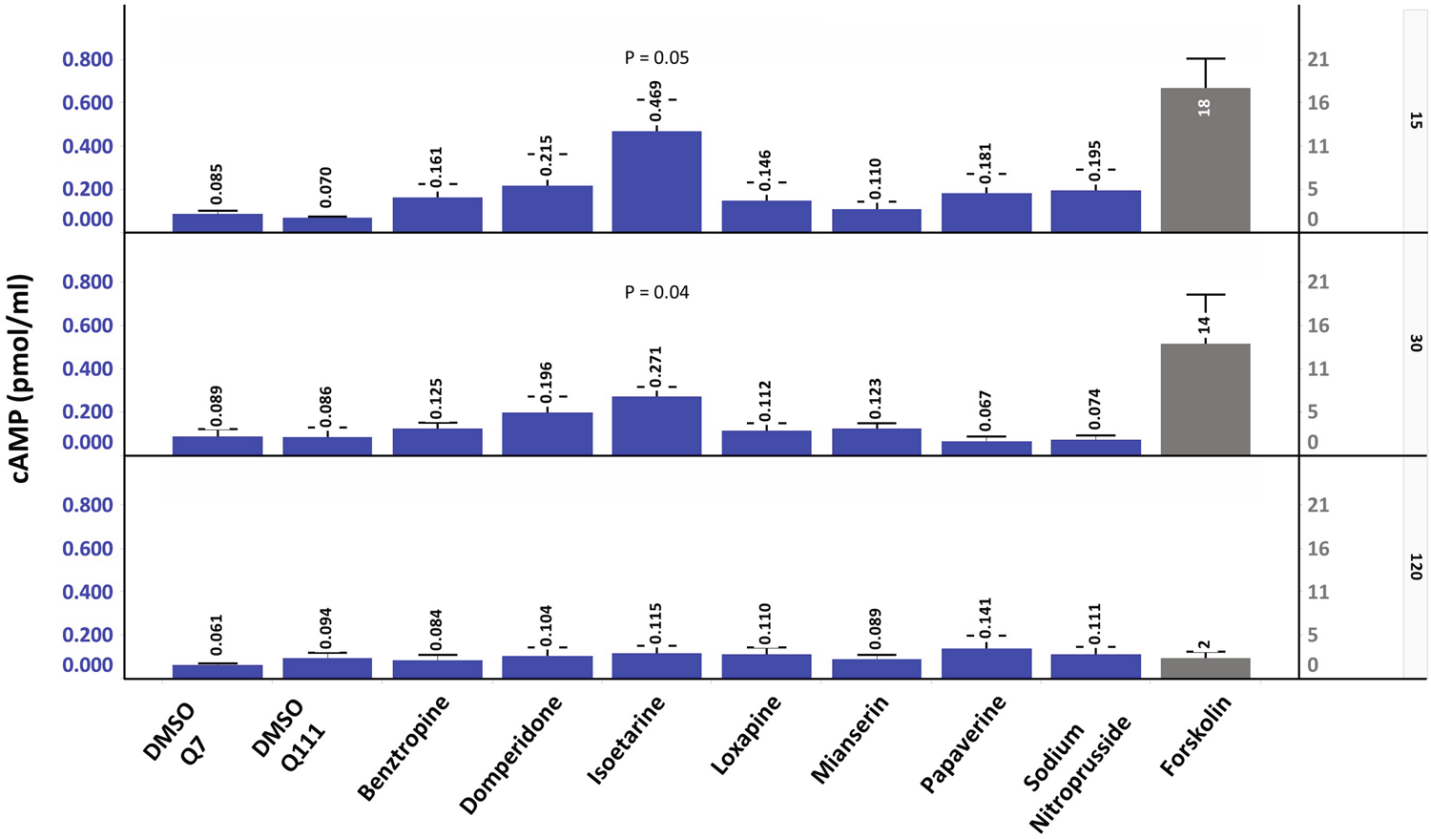
Protective compounds can induce cAMP. cAMP levels were determined in ST*Hdh* ^*Q111*^ cells after incubation with benztropine (25 μM), domperidone (6 μM), isoetarine (50 μM), loxapine (6 μM), mianserin (25 μM), papaverine (25 μM), and sodium nitroprusside (66 μM) for 15, 30, and 120 minutes. Though isoetarine was the only compound to show a statistically significant change at 15 and 30 minutes, except for mianserin, the other compounds showed at least a two-fold increase in cAMP levels at 15 mins. Over time the induced levels of cAMP decreased back to the control levels. Forskolin significantly induced cAMP levels at 15 and 30 minutes with the highest levels seen at 15 minutes. The values are the average from three independent experiments (+/−S.E.) except papaverine where n = 2. All compounds except forskolin are plotted on the blue scale on the left, while forskolin is plotted on the grey scale on the right. The three panel rows are 15, 30, and 120 minutes. T-test was used to assess changes in cAMP levels relative to the ST*Hdh* ^*Q111*^ cells treated with DMSO.

Because cAMP/PKA signaling is a key pathway involved in cell survival and has been implicated in the pathophysiology of HD^25^, we tested whether these synergistic compounds may be working through augmenting cAMP and activating PKA. We assessed the ability of benztropine, domperidone, isoetarine, loxapine, mianserin, papaverine, and sodium nitroprusside to modulate cAMP levels in the ST*Hdh* ^*Q111*^ cells. cAMP levels were measured 15, 30, and 120 minutes after initial compound treatment in the presence of serum, which paralleled the pre-treatment stage of the PI assay, as it was anticipated that cAMP induction would be a relatively rapid response. All compounds, except for mianserin, showed at least a 2-fold increase in cAMP over the DMSO control at 15 minutes, which returned to control levels within 2 hours (Fig. 6). Though only isoetarine showed a statistically significant increase in cAMP levels at 15 and 30 minutes, the overall profile of increased levels at 15 mins and the gradual decrease over time for all of the compounds suggested that a transient induction of cAMP did occur shortly after initial compound treatment. Sodium nitroprusside, which primarily acts through stimulating cGMP, also produced an increase in cAMP. This 2-fold increase in cAMP by the protective compounds contrasted the 250-fold increase in cAMP levels induced by forskolin. Interestingly, forskolin did not show up as a hit in the LOPAC screen, nor did it show any protective effects when subsequently tested as a control in the PI assay run in parallel with the cAMP analysis (data not shown).

**Figure 7:**
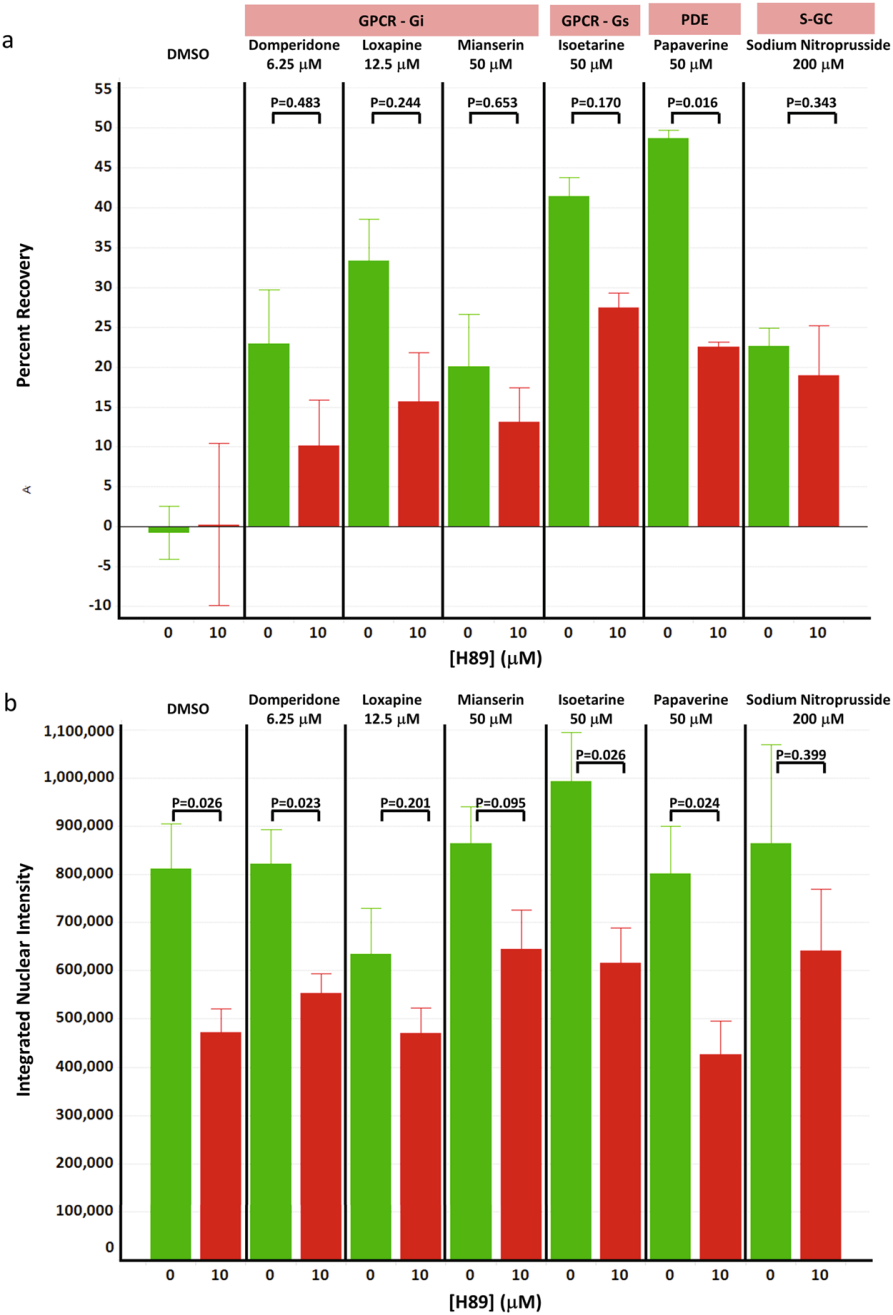
PKA inhibitor H89 inhibits the protective effects of several probes. (**a**) The protection of ST*Hdh* ^*Q111*^ cells from *mHTT* induced cell death by domperidone (6 μM), isoetarine (50 μM), loxapine (12.5 μM), mianserin (50 μM), papaverine (50 μM), and sodium nitroprusside (200 μM) co-incubated with the PKA inhibitor H89 (10 μM) was assessed in the 384-well PI assay. Benztropine (50 μM) was also tested, however, combination with H89 resulted in increased toxicity over the cell death seen in the DMSO control. The concentrations used were chosen to be on plateau of their respective activity curves (see Fig. 2). DMSO is H89 alone which showed no significant protection or toxicity. Analysis is from triplicate samples run in four independent experiments (Error bars are +/−SE). T-test was used to assess changes in the percent recovery levels relative to the ST*Hdh* ^*Q111*^ cells treated with compound without H89. While only papaverine showed a statistically significant decrease, the other compounds showed a trend for H89 inhibition of the protective effects. (**b**) The integrated intensity of the pCREB signal was measured in the nucleus of the ST*Hdh* ^*Q111*^ cells treated as above. CREB is a substrate for PKA and is used here as a surrogate marker for PKA activity to demonstrate inhibition of PKA activity by H89. Analysis is from triplicate samples run in four independent experiments (Error bars are +/−SE). T-test was used to assess changes in the pCREB intensity relative to the ST*Hdh* ^*Q111*^ cells treated with compound without H89.

To determine if PKA may be involved in the protective effect of these compounds, we incubated the ST*Hdh* ^*Q111*^ cells with benztropine, domperidone, isoetarine, loxapine, mianserin, papaverine, and sodium nitroprusside in the presence the PKA inhibitor H89 under the standard PI protection assay conditions. H89 has been used extensively in the literature as a selective and potent inhibitor of PKA^26,27^. If the protection from cell death by these compounds involved activation of PKA, then the addition of an inhibitor of PKA would be expected to reverse the protective effects of the compounds. Co-incubation of 10 μM H89 with the Gi-coupled GPCR antagonists domperidone, loxapine, and mianserin resulted in 56, 52, and 35 percent reduction, respectively, in the level of protection, while the Gs-coupled agonist isoetarine resulted in a 34 percent reduction, the PDE inhibitor papaverine a 55 percent reduction, and the s-GC agonist sodium nitroprusside a 17 percent reduction compared to compound alone (Fig. 7a). Since the primary canonical mechanism of sodium nitroprusside is activation of PKG, and given that H89 is ~10-fold selective for PKA over PKG, the absence of a marked effect with sodium nitroprusside is not unexpected. The relatively lower effect of H89 on the PKG activator sodium nitroprusside compared to the PKA activators is consistent with the canonical mechanisms of these compounds. To confirm inhibition of PKA activity by H89 under the conditions of the PI assay we measured the levels of nuclear pCREB using high content analysis (Supplementary Figure S4). Consistent with the heterogeneity seen in the response of the ST*Hdh* ^*Q111*^ cells to protection by the compounds, a heterogeneous distribution of pCREB levels was also detected (Supplementary Figure S5). The levels of pCREB were decreased in the presence of 10 μM H89 in all cases indicating inhibition of PKA activity (Fig. 7b). While H89 has been used extensively as a selective and potent inhibitor of PKA to understand the biology of PKA signal transduction, it has been reported that H89 has other effects as well^28^. To address this, we also tested the effects of PKI, a reportedly more selective PKA inhibitor, on the activity of these compounds, however, PKI by itself was toxic to the ST*Hdh* ^*Q111*^ cells which overshadowed any potential effect in inhibiting protection (data not shown).

To further assess PKA activation, we quantified the levels of PKA phosphorylated at threonine 197 (pPKA) in the catalytic subunit using high-content analysis. We examined the pPKA levels at 24 hours after serum free conditions since this was the condition where we measured the protection of the compounds. The levels of cytoplasmic pPKA were lower in the ST*Hdh* ^*Q111*^ cells relative to the ST*Hdh* ^*Q7*^ (Supplemental Figure S6), consistent with the hypothesis that elevated pPKA was associated with neuronal cell survival. Benztropine, isoetarine, loxapine, mianserin, and sodium nitroprusside exhibited a concentration-dependent increase in cytoplasmic pPKA approaching the levels of the wild type ST*Hdh* ^*Q7*^ cells. The concentration response for domperidone, papaverine and forskolin was less pronounced. In contrast to the cytoplasm, the nuclear pPKA levels in the ST*Hdh* ^*Q111*^ cells were higher than in the ST*Hdh* ^*Q7*^ cells (Supplemental Figure S6). None of the compounds showed a marked concentration-dependent decrease in the nuclear levels. The increase in cytosolic pPKA correlated with the percent recovery for these compounds (Supplementary Figure S7); however, the concentration response curves between the compounds were distinct from each other. If pPKA were the only factor responsible for the protective effects of these compounds, then the concentration response curves for the pPKA effect on recovery would be expected to be the same. The fact that they were different suggests additional mechanisms were involved in the protection phenotype for these compounds.

### Some compounds may be protecting by non-canonical mechanisms

**Figure 8:**
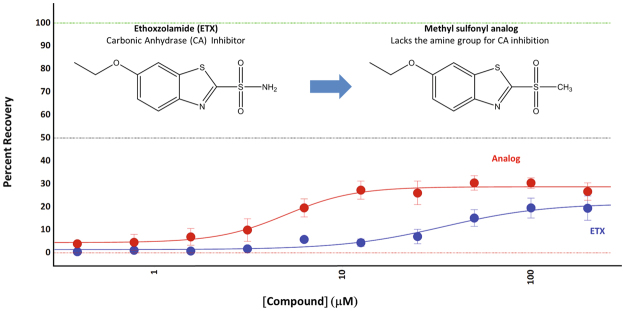
Ethoxzolamide may not work through the canonical carbonic anhydrase mechanism. The methyl sulfonyl analog of ETX does not contain the sulfonamide group of ETX and it is not expected to inhibit carbonic anhydrase^29^, though we did not test this directly. This analog is 7-fold more potent than ETX in protecting ST*Hdh* ^*Q111*^ cells from stress induced cell death in the propidium iodide assay suggesting that the mechanism of protection of ETX is not through carbonic anhydrase inhibition. Acetazolamide, brinzolamide and dorzolamide, all reported carbonic anhydride inhibitors, did not protect ST*Hdh* ^*Q111*^ cells (see Supplementary Figure S8) further supporting the idea that inhibition of carbonic anhydrase is not a protective mechanism. Interestingly, the methyl sulfonyl analog only protected ~50% of the ST*Hdh* ^*Q111*^ cells consistent with the existence of distinct protection mechanisms in different subpopulations of cells.

Our pathway analysis was based on using canonical mechanisms of action for the identified compounds; however, we hypothesized that the protective activity of some of the compounds might be through alternative mechanisms, as well. Several structurally distinct carbonic anhydrase inhibitors were present in the library of compounds, but only one of them, ethoxzolamide, showed protective activity in the PI assay (Supplementary Figure S8). To determine if ethoxzolamide was acting through its canonical carbonic anhydrase inhibition mechanism, we synthesized its methyl sulfonyl analog in which the amine group that is critical for the carbonic anhydrase inhibition by this drug class^29^ was replaced by an isosteric methyl group. We demonstrated that the methyl sulfonyl analog of ethoxzolamide was approximately 7-times more potent than ethoxzolamide itself and equally efficacious (Fig. 8). Though the methyl sulfonyl analog for inhibition of carbonic anhydrase was not tested directly, the activity of the methyl sulfonyl analog suggests that the protective activity observed with ethoxzolamide may be due to a distinct mechanism and not due to its canonical carbonic anhydrase inhibition.

## Discussion

Despite major technological advances in genome editing, differentiation of patient-derived iPSCs, and recapitulation of complex disease phenotypes in human microphysiological models (i.e., organs-on-a -chip), our knowledge of disease mechanism is often the limiting factor for optimizing therapeutic strategies for patient cohorts. QSP has emerged as an approach to address this void^10,11^. Commensurate with advances in the development of clinically relevant models, and complementary to systematic genetic approaches^30^, we anticipate an increased use of mechanistically diverse and well annotated chemical libraries, especially those containing FDA approved drugs, to probe disease mechanism. This small molecule approach has the potential to lead *directly* to drug repurposing and optimal drug combination strategies that maximize efficacy and minimize toxicity, as well as to serve as a starting point for selecting targeted libraries for additional discovery efforts. Thus, we expect that this approach will play an increasingly important role in mechanistic studies and drug development efforts to address many of the 7,000 rare diseases that exist worldwide. In the case of HD, screening identified several drugs having well-defined canonical modes of action that partially protected against mutant *HTT*-induced neuronal cell death. The fact that only the mutant cell line shows cell death under the stress conditions demonstrates that this phenotype is disease dependent, and the fact that the compounds are protective in the mutant cell line indicates that they are active in reversing the disease dependent phenotype. Many combinations exhibited significant synergy, suggesting a functional network association among them involving PKA (PKG) signaling.

The analysis reported here suggested that cAMP/PKA signaling was involved in the protection of neuronal cells from *mHTT*-induced toxicity in the ST*Hdh* ^*Q111*^ model. Several lines of evidence from the literature suggest that altered activity of the PKA (PKG) signaling is directly pathogenic and does not simply represent a beneficial compensatory mechanism for averting *mHTT*-induced cell death. Single cell analysis employing an optical pulse-chase method^31^ has demonstrated that neuron-to-neuron variation in protein homeostasis capacity (i.e., proteasome activity) contributes substantially to a given cell’s susceptibility to the effects of misfolded proteins^31^. Specifically pertinent to HD, striatal neurons were, on average, more vulnerable to disease-causing misfolded *mHTT* and cleared a corresponding -*mHTT* reporter more slowly than cortical and cerebellar neurons. Statistical modeling linked intrinsic protein homeostasis capacity in striatal, cortical, and cerebellar neurons to their vulnerability to *mHTT*-induced degeneration. Furthermore, animal models of HD show that *mHTT* stress-induced impairment of the proteasomal capacity in the striatum is associated with lowered PKA activity^25^. This reduced PKA activity is caused by the accumulation of negative regulatory PKA subunits that are normally controlled by proteasomal degradation. Since it has also been shown that full proteasomal activity depends upon PKA phosphorylation, a feed-forward loop of diminished PKA and proteasomal activity has been suggested as an important component of HD pathogenesis. Consistent with the results presented here, pharmacologic intervention corroborated this hypothesis, as agents that increase cAMP and activate PKA restored proteasomal activity and ameliorated motor impairment^31^. By analogy, very recent results indicate a similar feed-forward loop operative in other tauopathies^32^. Our results showing the inhibition of the protective activity of the compounds by a PKA inhibitor, a lower level of cytosolic pPKA in the *mHTT* cells relative to the *wt* cells under stress conditions, and the association of increasing pPKA with increasing recovery from cell death are consistent with the observations in the literature. The lack of a marked increase in pPKA by domperidone or papaverine does not necessarily contradict the observation that the PKA inhibitor H89 prevented protection by these compounds. The spatiotemporal activation and regulation of cAMP and PKA is complex^33–37^ and the 24 hour time point may not have been optimal to capture activation by all of the mechanisms. However, the fact that the PKA recovery curves were different among the compounds suggests that factors in addition to activation of PKA per se may also contribute to neuronal cell protection.

Forskolin also increased pPKA to levels that were associated with protection by the other compounds yet itself was not protective, further suggesting that additional factors are important for protection. Since forskolin was unable to induce protection from cell death in ST*Hdh* ^*Q111*^ cells, it appears that regulatory nuances beyond simply a global and robust stimulation of cAMP downstream of specific GPCR machinery are necessary to elicit a protective response. While the inability of forskolin to protect could result from its well-known off-target effects (e.g., glucose transporter^38^), strong nonselective stimulation of cAMP could result in antagonistic combinatorial effects consistent with our results showing that the majority of combinations of partially protective compounds were indeed antagonistic (or toxic). On the other hand, an intrinsic characteristic of cAMP/PKA signaling is compartmentalization, and subcellular localized generation of cAMP is tightly coupled to activation of PKA^33–35,37^. Therefore, it is tempting to speculate that crosstalk between two cAMP/PKA compartments could provide the basis for the observed synergy between two compounds acting along the cAMP/PKA signaling axis and result in the necessary spatial and temporal modulation of cAMP/PKA signaling to elicit a protective response. We expect that extension of the imaging analysis initiated in this study in conjunction with additional cAMP/PKA signaling biosensors will enable the role of signaling compartmentalization in the protection from the pleiotropic effects of mutant HTT to be determined and perhaps offer insights into the mechanistic underpinnings of the pathogenic dysregulation.

We found that the canonical targets of a number of compounds converge on a plausible mechanism for neuroprotection from *mHTT* toxicity, and that the literature supports the role of this mechanism in HD. However, this mechanism alone neither explains all of our results nor provides a clear path to an HD therapeutic. Given the pleiotropic nature of *mHTT*, and evidenced by our synergistic results that do not involve cAMP/PKA signaling, we anticipate that other protective mechanisms exist. Further, although the present work focuses on within-pathway convergence as the mechanism of synergy, it is also possible that the synergistic effects that we see result from mechanistic heterogeneity within the cellular population. Addressing this possibility could provide insight into the basis for distinct vulnerabilities among subpopulations of mHTT-expressing cells and the relationships among the different pathways regulating their susceptibility to stress-induced cell death. In the next iteration of the QSP cycle, we are broadening the analysis to obtain a more complete picture of pathways and networks involved in the protection of the Q111 cells based on the canonical mechanisms of active probes. In addition, the canonical mechanisms may not be the only mechanisms through which compounds protect from *mHTT* toxicity, as exemplified by the activity of the ethoxzolamide analog. Although *mHTT* is pleiotropic, small molecule compounds can also interact with multiple targets; it has been estimated that most drugs bind to on average 6 targets^39^. Indeed, modulation of non-canonical targets in addition to activation of the PKA pathway by the seven probes could help explain why we see only partial inhibition of recovery by H89. Exploring non-canonical mechanisms has the potential to lead to the identification of novel pathways for neuronal cell protection and emphasizes the value of assembling chemical libraries containing structurally distinct probes that have the same canonical mechanism. Thus, in subsequent iterations of the QSP analysis we are applying various approaches^40^ including chemical proteomics^41,42^ to identify the targets to which the protective compounds are binding, and computationally expanding the potential targets and pathways to predict non-canonical interactions of the protective compounds. We are also actively expanding the scope of potential mechanisms by analyzing additional synergistic neuronal cell protective pairs and screening larger mechanistically annotated libraries (e.g. NCATS Pharmacologically Active Chemical Toolbox library). Key to this whole approach is the systems level analysis that ensures a mechanistically unbiased assessment of the biology, which will enable more efficient and novel approaches to therapeutic design in the long run.

The work presented here represents the first two iterations of the QSP platform approach developed at the University of Pittsburgh^10^ starting with mechanism-annotated probe compounds and a clinically relevant phenotypic assay, and leading to the identification of disease-relevant pathways. We show that an integrated chemogenomic strategy using information about probes that modulate a clinical phenotype can lead to testable hypotheses and provide insights to targetable biological mechanisms for disease treatment. To our knowledge, this is the first report of such an approach applied to HD. This initial chemogenomics analysis can be extended to include medium spiny neurons derived from human iPSC in the context of human neuronal microphysiological systems^43^ that recapitulate critical cell intrinsic and extrinsic microenvironments. Further, we are developing a comprehensive computational model of disease progression through the integration of the chemogenomic analysis and transcriptomic profiles of HD in both mouse and human tissues, which will enable refinement of testable hypotheses. Building on the analyses reported here, additional iterations of experimentally testing hypotheses and refining models will lead to emergent properties of HD disease and therapeutic strategy design. While the focus here is on HD the approach described can be broadly applied to any discovery strategy involving small molecule modulation of a disease phenotype.

## Materials and Methods

### Cells

Conditionally immortalized mutant huntingtin (*mHTT*) homozygous knock-in mouse ST*Hdh* ^*Q111*^ cells and the isogenic wild type ST*Hdh* ^*Q7*^ cells were a gift from Marcy MacDonald and are described in Trettel *et al*.^13^. Cells were cultured in DMEM (25 mM glucose, 4 mM L-glutamine) supplemented with 10% FBS, 5 mM sodium pyruvate and 0.3% Pen-Strep at 33 °C and 5% CO_2_. The stress conditions for testing compounds involved incubation of the cells in serum-free medium at 37 °C and 5% CO_2_.

### Compound Preparations

The LOPAC library and individual test compounds were from Sigma-Aldrich (St. Louis, MO) except for meclizine, prednisolone, and ethoxzolamide which were from Santa Cruz (Dallas, TX), and U83836E which was from Abcam (Cambridge, MA). Compounds were dissolved in DMSO (Alpha Aesar, Fisher Scientific) to 10 mM stocks. For screening, the LOPAC library was first diluted in medium containing 5% DMSO and then 5 μl were added to 45 μl of cells. For concentration response curves, 2- or 3- step serial dilutions in DMSO were prepared, a 20-fold intermediate dilution was made in medium, and 5 μl of this solution were added to cells in 45 μl medium. For combination treatments, compounds were mixed together in DMSO before being diluted in medium as above for addition to the cells.

### Synthesis of 6-ethoxy-2-(methylsulfonyl) benzo[d]thiazole

Reagents for the synthesis of the methylsulfonyl analog of ethoxzolamide were purchased from Sigma-Aldrich (St. Louis, MO). The synthesis was performed in two steps as follows:
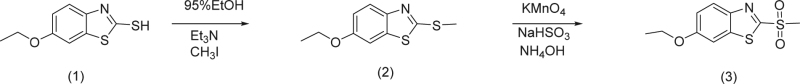


Synthesis of 6-ethoxy-2-(methylthio) benzo[d]thiazole (**2**)
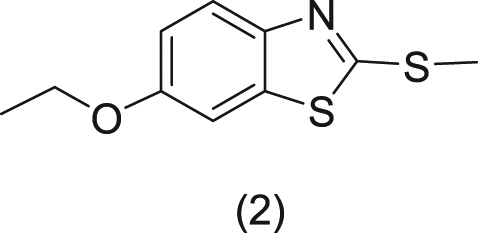


Starting with 6-ethoxybenzo[d]thiazole-2-thiol (1) from Sigma-Aldrich, we followed the protocol described by Rosen *et al*.^44^ to synthesize (2).

Synthesis of 6-ethoxy-2-(methylsulfonyl) benzo[d]thiazole (**3**)
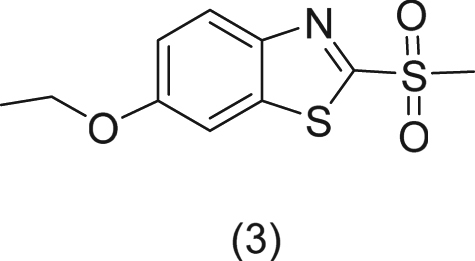


To a solution of 6-ethoxy-2-(methylthio) benzo[d]thiazole **(2)** (0.5 g, 2.219 mmoles) dissolved in acetic acid (6 mL)was added KMnO4 (0.596 g, 3.772 mmoles) in water (8 mL). The resulting mixture is stirred at room temp for 5 days. The reaction mixture was quenched with NaHSO_3_ (0.346 g, 3.33 mmoles) and the pH of the solution adjusted to 8 with NH_4_OH. The reaction mixture was then extracted with EtOAC. Organic solvents washed with water, brine dried over sodium sulfate and the solvents evaporated to give 0.5 g (87%) of **(3)** as a white solid. (^1^HNMR (400 MHz, DMSO-d6) δ 1.39(t, 3 H, *J* = 7.2 Hz), 3.54(s, 3 H), 4.15(q, 2 H, *J* = 7.2 Hz), 7.29(dd, 1 H, J = 8.8 Hz & 2.4 Hz), 7.87(d, 1 H, *J* = 2.4 Hz), 8.13(dd, 1 H, *J* = 8.8 Hz & 0.4 Hz) AT-IR cm^−1^ 2981, 2920, 1597, 1553, 1486, 1471, 1402, 1312, 1250, 1228, 1139, 1116, 1068, 1037, 1018, 963, 935, 883, 858, 816, 760, 690, 610. HRMS (TOF MS Ap+) m/z calcd for C_10_H_12_NO_3_S_2_ (258.0259) Found (258.0265)).

### 384-well PI assay

Cells were cultured for 4 days in complete medium at 33 °C and 5% CO_2_ before plating in Greiner Bio-One TC, clear bottom, black walled 384-well plates (50 μl) at 3,000 cells per well. Cells were allowed to attach in complete medium at 33 °C and 5% CO_2_ for 24 hr. Prior to compound treatment, medium was removed and the cells washed once with PBS (pH = 7.4) after which 45 μl of complete medium was added. Compounds in 5 μl complete medium were added to cells. Cells were incubated at 33 °C and 5% CO_2_ for 2 hrs after which they were washed twice with PBS and 45 μl of serum free medium was added. Compounds in serum free medium (5 μl) were added and cells were incubated at 37 °C and 5% CO_2_. After 24 hrs, 25 μl of PBS containing 15 μg/ml Hoechst 33342 and 12 μg/ml propidium iodide (PI) were added and cells were incubated for 30 min at room temperature on a rocker protected from light.

### 384-well High Content Screening (HCS) and Analysis

Images were collected on the ImageXpress Ultra (IXU) sequentially acquiring Hoechst (Ch1, 405/447 nm) and Texas Red (Ch2, 561/685 nm) using a 10x Plan Fluor objective. Image analysis was carried out using the Multiwavelength Cell Scoring application in the MetaXpress software. The nuclear compartment was identified in Ch1 with a threshold intensity of 2,000 above background and the Texas Red compartment was identified in Ch2 with a threshold intensity of 20,000 above background.

Heterogeneity analysis of the distribution of PI labeling in the ST*Hdh* ^*Q111*^ cell population was done using the heterogeneity indices described by Gough *et al*.^14,45^. The combined assessment of the population diversity (quadratic entropy (QE)), non-normality (Kolmogorov-Smirnov (KS)) and percent outliers classified the control populations as having macro-heterogeneity, and review of the HistoBox plot indicated a bimodal distribution. Thus, all subsequent analysis of the images was performed at the cell level and population average measurements were not used.

For the analyses presented here the Percent Recovery was calculated from the Percent PI positive cells by:

1 $$(1-\frac{(\mathrm{Percent}\,\mathrm{PI}\,\mathrm{Positive}\,\mathrm{cells}\,\mathrm{for}\,\mathrm{Cpd})\,-\,(\mathrm{Percent}\,\mathrm{PI}\,\mathrm{Positive}\,\mathrm{cells}\,\mathrm{for}\,\mathrm{Positive}\,\mathrm{Ctrl})}{(\mathrm{Percent}\,\mathrm{PI}\,\mathrm{Positive}\,\mathrm{cells}\,\mathrm{for}\,\mathrm{Negative}\,\mathrm{Ctrl})\,-\,(\mathrm{Percent}\,\mathrm{PI}\,\mathrm{Positive}\,\mathrm{cells}\,\mathrm{for}\,\mathrm{Positive}\,\mathrm{Ctrl})})\times 100$$

where positive controls are the ST*Hdh* ^*Q7*^ cells, and negative controls are the ST*Hdh* ^*Q111*^ cells treated with DMSO.

### 384-well LOPAC library screen

The LOPAC library was screened in the 384-well PI assay at 30, 10, and 3 μM. Compounds that showed increased percent recovery at least 3 SD above the mean of the ST*Hdh* ^*Q111*^ DMSO control and were not toxic, having a total cell number that was not less than 3 SD below the total cell number of the ST*Hdh* ^*Q111*^ DMSO control, were picked for confirmation in a concentration response assay.

### cAMP assay

cAMP measurements were performed using the cAMP Enzyme Immunoassay kit (CA-200, Sigma-Aldrich, St Louis, MO, USA), following the acetylated version of protocol supplied by the vendor. Cells were plated in 96-well plates at 9,000 cells per well and allowed to attach in complete medium at 33 °C and 5% CO_2_ for 24 hr. Cells were treated with 10 μl DMSO or compounds and incubated in complete medium at 33 °C and 5% CO_2_ for 15, 30, and 120 minutes at which time the medium was removed and the cells were lysed with 250 μl of 0.1 N HCl for 20 minutes. Equivalent amounts of samples were used in the ELISA assay as determined by protein concentration. Protein concentrations were determined using the Bradford protein assay (Bio Rad).

### PKA inhibition analysis

Cells were treated with benztropine, domperidone, loxapine, mianserin, isoetarine, papaverine, and sodium nitroprusside either alone or in the presence of 10 μM H89 (Sigma-Aldrich St. Louis, MO) following the treatment protocol for the 384-well PI assay described above. Plates were imaged and analyzed as described in the 384-well High Content Screening (HCS) and Analysis above. Following the live cell image acquisition, the plates were fixed with 3.7% formaldehyde in PBS and permeabilized with 0.1% Triton X-100. Plates were blocked with 4% normal goat serum for 1 hr and then incubated overnight with rabbit anti-pCREB (Ser133, Cell Signaling Technology, Danvers, MA) at 4 °C. Plates were wash three times with 1x PBS and incubated with Alexa-Fluor 488-conjugated Goat anti-Rabbit IgG (H + L) (Jackson ImmunoResearch, West Grove, PA) for 1 hr at room temperature, washed three times with 1x PBS, and imaged. Images were collected on the IXU sequentially acquiring Hoechst (Ch1, 405/447 nm) and Alexa 488 (Ch2, 488/514 nm) using a 10x and 40x objectives. Image analysis was carried out using the Multiwavelength Cell Scoring application in the MetaExpress software. The nuclear compartment was identified in Ch1 with a threshold intensity of 2,000 above background (the nuclear mask) where the FITC intensity was measured.

### PKA activation assay

Cells were treated as described for testing in the 384-well PI assay. At 24 hr the cells were fixed with 3.7% formaldehyde in PBS with 4 µg/ml Hoechst 33342 and permeabilized with 0.1% Triton X-100. Plates were blocked with 3% BSA in PBS and were incubated with rabbit anti-PKA (αβγ catalytic subunit; EP2606Y; phospho-T197, abcam, Cambridge MA) mAb overnight at 4 °C. Plates were wash three times with 1x PBS and incubated with Alexa-Fluor 488-conjugated Goat anti-Rabbit IgG (H + L) (Jackson ImmunoResearch, West Grove, PA) for 1 hr at room temperature, washed three times with 1x PBS, and imaged. Images were collected on the IXU sequentially acquiring Hoechst (Ch1, 405/447 nm) and Alexa 488 (Ch2, 488/514 nm) using a 20x (0.45-NA) ELWD objective. Image analysis was carried out using the Multiwavelength Translocation application in the MetaExpress software. The nuclear compartment was identified in Ch1 with a threshold intensity of 5,000 above background (the nuclear mask). The nuclear mask was eroded 1 µm to create the Inner region mask in Ch2. The nuclear pPKA in each cell was measured as the Mean Inner Intensity Ch2 of the Alexa 488 label within the Inner region mask in Ch2. The cytoplasmic pKA was measured in a 3 μm wide ring around the nuclear mask.

### 1536-well PI Assay

ST*Hdh* ^*Q111*^ or ST*Hdh* ^*Q7*^ mouse striatal cells were plated in black wall, clear bottom 1536-well cyclic olefin polymer-type imaging plates (Edition Eight; Whitefish, MT) at 1.2 × 10^3^ cells per well in 5 µl volume using a Multidrop Combi Reagent Dispenser (ThermoFisher). Growth medium was DMEM (25 mM D-glucose; ThermoFisher) supplemented with 1% fetal bovine serum (Hyclone), 5 mM sodium pyruvate (ThermoFisher), and 0.3x penicillin/streptomycin (ThermoFisher). Cells were incubated for 16 h in a humidified incubator maintained at 33 °C and 5% CO_2_. 46 nl of compounds (NCATS Pharmaceutical Collection or vehicle control; qHTS format; 5 concentrations spanning 30 nM–50 μM) were transferred using a Kalypsis pin tool and plates were returned to 33 °C for 2 h. Cells were moved to a humidified incubator maintained at 37 °C and 5% CO_2_ for 24 h. Hoechst 33342 (ThermoFisher) and propidium iodide (PI, Sigma-Aldrich) were prepared in PBS and 1 µl was added to each well, yielding a final concentration of 4 µg/ml and 5 µg/ml, respectively. Plates were incubated at room temperature for 30 min prior to imaging.

### 1536-well Imaging and Analysis

Plates were imaged on an IN Cell 2200 widefield automated microscope (GE Healthcare) using a 10 × 0.45 NA air objective and standard DAPI (390/18x, 432/48 m) and Cy3 (542/27x, 587/45 m) filter sets, both at 30 milliseconds of exposure. One field of view per well, encompassing the entire well was chosen for imaging. Digital images were analyzed using IN Cell Analyzer Workstation Software v3.7.3 (GE Healthcare) with the Multi Target Analysis canned analysis protocol. Briefly, Hoechst nuclei were identified using top hat segmentation (objects with minimum area of 75 µm and a sensitivity setting of 87). All available data parameters were captured on a cell by cell basis for both nuclei and PI objects. A mean PI intensity of more than 3 STDEV above the background mean was classified as PI positive. Data were normalized to controls on a per-plate basis (Q7 + vehicle and Q111 + vehicle), with percent recovery calculated as above (equation 1). Concentration-response curves were generated using NCATS software (https://tripod.nih.gov/curvefit/) and active compounds had curve-class designations of 1.1, 1.2, 2.1, and 2.2^46^.

### Drug combination analysis

For single concentration combination experiments, compounds were mixed together in pairs using concentrations that were at or near the plateau of the respective concentration curves for the individual compounds. Activity of the combinations was assessed in the 384-well assay described above. The Bliss combination index was calculated using the Bliss Independence Model:

2 $$Bliss\,Combination\,Index=RF12/((RF1+RF2)-(RF1\,\ast \,RF2)/100)$$

where RF12 is the percent recovery of the combination of compounds 1 and 2, RF1 = the percent recovery of compound 1, and RF2 is the percent recovery of compound 2.

For selected compounds, the effect of compounds paired in a concentration response curve was assessed by mixing compounds at four concentrations each. Two concentrations used were on the respective plateaus of the single compounds, and two were on the slope before the plateau. The combination index was calculated using the method of Chou and Talalay^20,47^ and isobolograms were drawn in Spotfire (Tibco, Boston, MA). The Chou-Talalay Median-Effect model accounts for the dose response of drugs to determine the combination effect. The resultant equation for the model is as follows:

3 $$CI=\frac{{D}_{1}}{{D}_{x1}}+\frac{{D}_{2}}{{D}_{x2}}$$

where *D*_1_ and *D*_2_ denote the doses of compound 1 and compound 2 required to reach an effect of *x*% as single treatment, while *D*_x__1_ and *D*_x__2_ are the doses needed in combination to inhibit *x*%, respectively. Combinations were examined for induction of antagonism (CI > 1.1), additivity (0.9 < CI < 1.1), synergy (CI < 0.9) and strong synergy (CI < 0.3).

### Computational predictions of drug-target binding

We identified 83 compounds as potentially neuroprotective using a latent factor model (LFM) combined with structural similarity. Our LFM approach, Balestra^48,49^, is based on probabilistic factorization of the incomplete drug-target interaction matrix. Given a binary matrix, **R**, of interactions between *N* drugs and *M* targets, Balestra decomposes it into the product of two matrices, **U** and **V**, that express the drugs and targets in terms of *D* latent variables,

4 $${{\bf{R}}}_{N\times M}={{\bf{U}}}_{N\times D}^{T}{{\bf{V}}}_{D\times M}.$$

This decomposition assigns values – loosely comparable to interaction probabilities – to the previously undetermined elements of **R**. Our LFM was trained on chemical-target interaction data from DrugBank (version 4.0.0, approved drug subset) and STITCH (version 3.0, experimental data only) databases. We identified from the same databases all canonical targets of 15 hit compounds from an earlier mitochondrial screen^6^ and 9 compounds that are in clinical trials for neuroprotection in HD. Compounds that the LFM predicted to have interaction values greater than 0.9 were selected as potentially neuroprotective. In addition to the LFM, the ROCS module in OpenEye software^50^ was used to predict neuroprotective compounds based on 3D structural similarity. A separate query was built based on the 3D shape and heavy atom properties of each of the 15 compounds from the mitochondrial screen. Each query was used to search compounds in DrugBank, and the top ranked compounds were selected based on the OpenEye ComboScore measure of shape and atom properties. The final set of predicted neuroprotective compounds was generated by merging the results from LFM prediction and 3D structural similarity search.

### Pathway analysis

All canonical targets for the probes that showed cell protection were identified in DrugBank (version 4.5.0, approved drug subset) and STITCH ligand-protein interaction database (version 4.0, human subset with an experimental confidence score greater than 0.7), as well as data mining from the literature. The 22 probes were mapped to 75 targets and detailed drug-target interaction mapping was shown in detail in Supplementary Table S2. Each target, and each probe by association, was then mapped to one or more pathways in the KEGG pathway database (http://www.kegg.jp, version 07, 2016, homo sapiens), ending up with 34 pathways as shown in Supplementary Table S3. We identified for further analysis all synergistic pairs of compounds in which the two compounds had different targets on the same pathway.

Over-representation of pathways among synergistic pairs in our screen is quantified using the enrichment factor

5 $$E{F}_{i}=\frac{\tfrac{{N}_{pair{s}_{i}}}{{N}_{pairs}}}{{(\tfrac{{N}_{compound{s}_{i}}}{{N}_{compounds}})}^{2}},$$

where ${N}_{pair{s}_{i}}$ is the number of synergistic pairs mapped into pathway i, ${N}_{pairs}=61$ is the total number of synergistic pairs identified in our combination screen, ${N}_{compound{s}_{i}}$ is the number of compounds from DrugBank and STITCH that mapped into pathway *i*, and ${N}_{compounds}$ is the total number of compounds we used from DrugBank and STITCH. The enrichment factor of a pathway is its propensity to be targeted by synergistic compound pairs in our screen.

### Data availability

Data are available from the authors upon reasonable request.

## Electronic supplementary material

Supplementary Information
