# Supplementary material for: Connecting Neuronal Cell Protective Pathways and Drug Combinations in a Huntington’s Disease Model through the Application of Quantitative Systems Pharmacology
Source: Sci Rep. 2017 Dec 19;7:17803. doi: 10.1038/s41598-017-17378-y (PMC5736652; doi:10.1038/s41598-017-17378-y)
Supplement: Supplementary file 1 — Supplementary Information [file 41598_2017_17378_MOESM1_ESM.pdf]

Connecting Neuronal Cell Protective Pathways and Drug Combinations in a Huntington's Disease Model through the Application of Quantitative Systems Pharmacology

Fen Pei[1], Hongchun Li[1], Mark J. Henderson[2], Steven A. Titus[2], Ajit Jadhav[2], Anton Simeonov[2], Murat Can Cobanoglu[1], Seyed H. Mousavi[3], Tongying Shun[4], Lee McDermott[5], Prema Iyer[5], Michael Fioravanti[5], Diane Carlisle[3], Robert M. Friedlander[3,6], Ivet Bahar[1,4], D. Lansing Taylor[1,4,6], Timothy R. Lezon[1,4,‡], Andrew M. Stern[1,4,‡], Mark E. Schurdak[1,4,‡,*]

[1]Department of Computational and Systems Biology, 3501 Fifth Ave, Suite 3064, Biomedical Science Tower 3, Pittsburgh, PA 15260.

[2]National Center for Advancing Translational Sciences, National Institutes of Health, 9800 Medical Center Drive, Rockville, MD 20850.

[3]Department of Neurological Surgery, 200 Lothrop St., UPMC Presbyterian, Suite B-400, Pittsburgh, PA, 15261.

[4]University of Pittsburgh Drug Discovery Institute, 200 Lothrop St., W950 Biomedical Science Tower Pittsburgh, PA, 15261.

[5]Department of Pharmaceutical Science, 3501 Terrace St., Pittsburgh, PA, 15261.

[6]University of Pittsburgh Brain Institute, 4074 Biomedical Science Tower 3, 351 Fifth Ave., Pittsburgh, PA, 15261.

*Corresponding author
Mark E Schurdak
Email: mes234@pitt.edu

‡Co-Senior authors

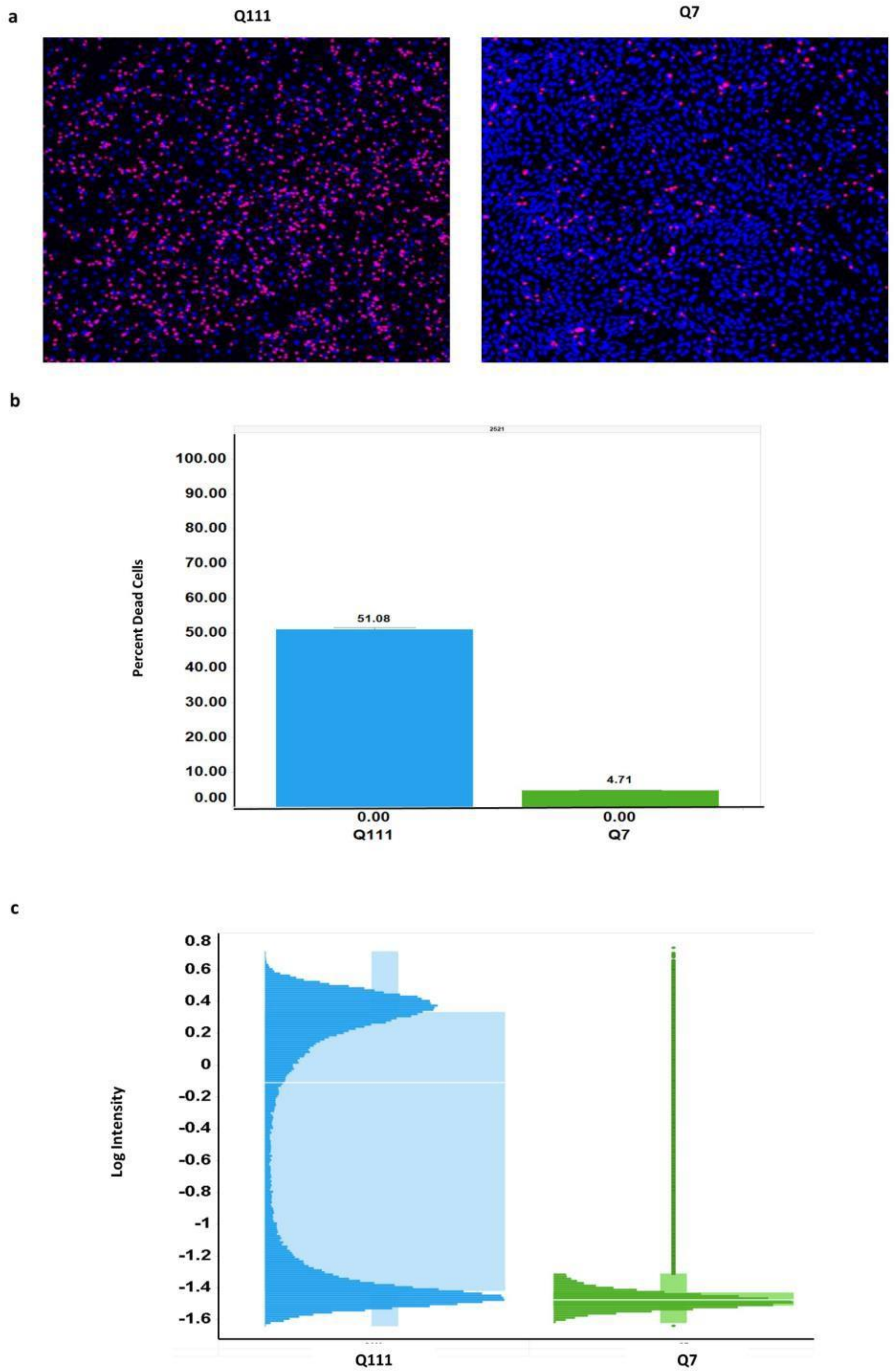

Figure S1. Heterogeneity in mHTT induced neurotoxicity in ST*Hdh*^{Q111} cells. a) ST*Hdh*^{Q111} and ST*Hdh*^{Q7} cells were incubated under serum free conditions for 24h at 37°C, labeled with Hoechest (blue) and PI (red) and imaged. b) Under stress conditions only ~ 50% of the ST*Hdh*^{Q111} cells die as evident by only half of the Hoechst positive cells labeling with PI. Only about 5-7% of the ST*Hdh*^{Q7} cells die under these conditions. c) Histograms quantifying the intensity of PI in the nucleus of ST*Hdh*^{Q111} cells (left) and ST*Hdh*^{Q7} cells (right).

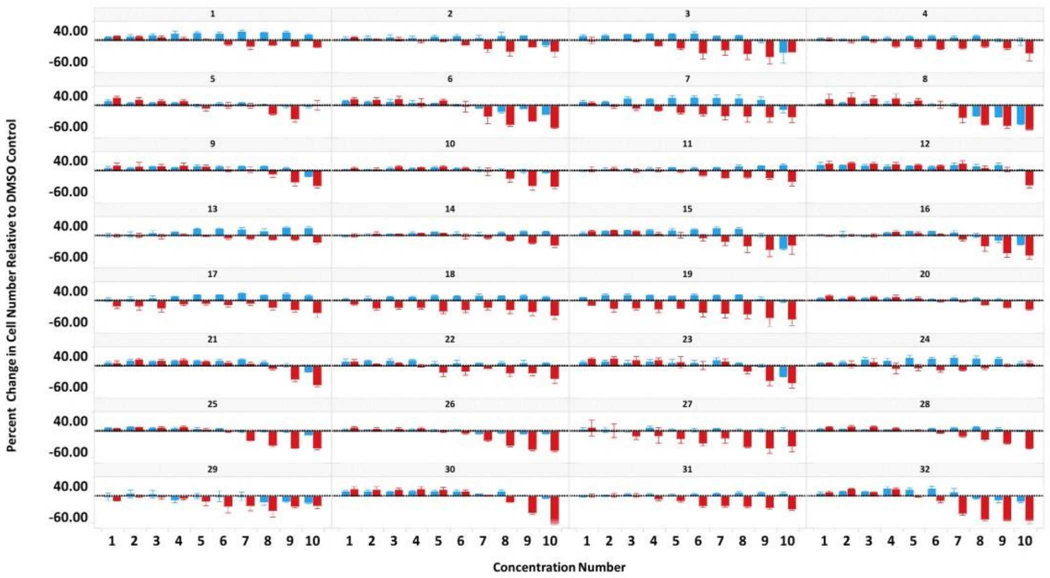

Figure S2. Change in total and dead cell numbers with compound treatment relative to DMSO. A greater percent change in the number of dead cells (PI positive, red bars) was seen compared to the change in total cell number (blue bars) indicating that the decrease in dead cell number was not simply due to loss of cells from the plate. Panel numbers are the compounds listed in Figure 2.

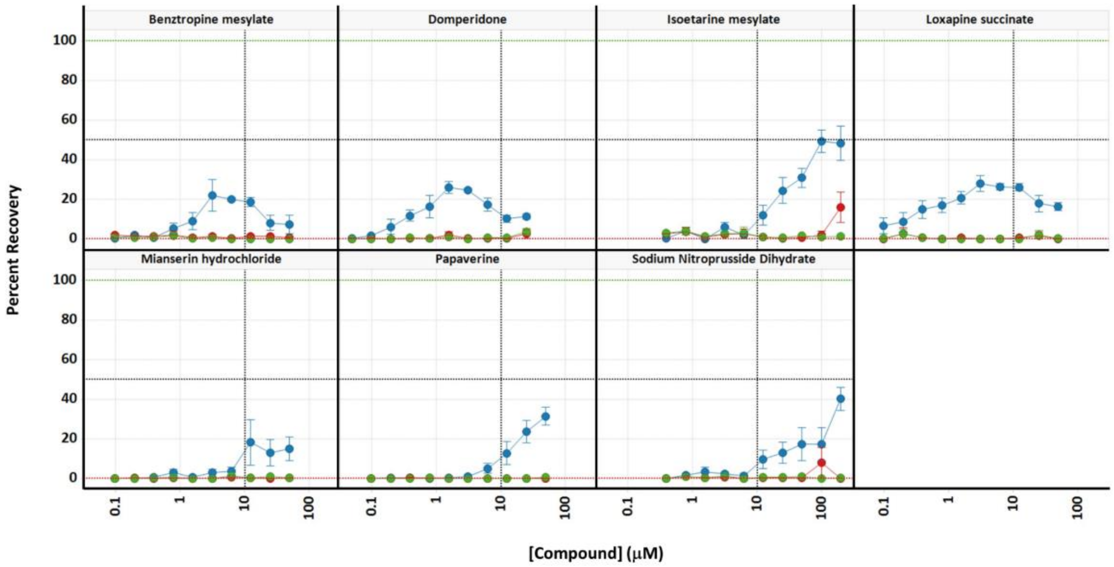

Figure S3. Protective compounds are not quenching the PI signal. Two sets of ST*Hdh*^{Q111} cells were treated with a compound titration (set 1) or DMSO (set 2) as per the standard 384-well protocol. At 24 hrs, both sets were labeled with PI and imaged. After imaging, to set 2 was added the compound titration and incubated at room temperature for 30 minutes and then imaged. The Percent Recovery was calculated for both sets. The blue curves show the compound titration curves of set 1 where the compounds were added before the PI. The red curves are the DMSO curves in set 2 before compound addition, and the green curves are the compound titration added to set 2 after PI addition. The compounds did not show the characteristic response curve when added after the PI indicating that the Percent Recovery seen with the compounds was not due to quenching of the PI signal. The analysis is from three independent runs (+/- S.E.).

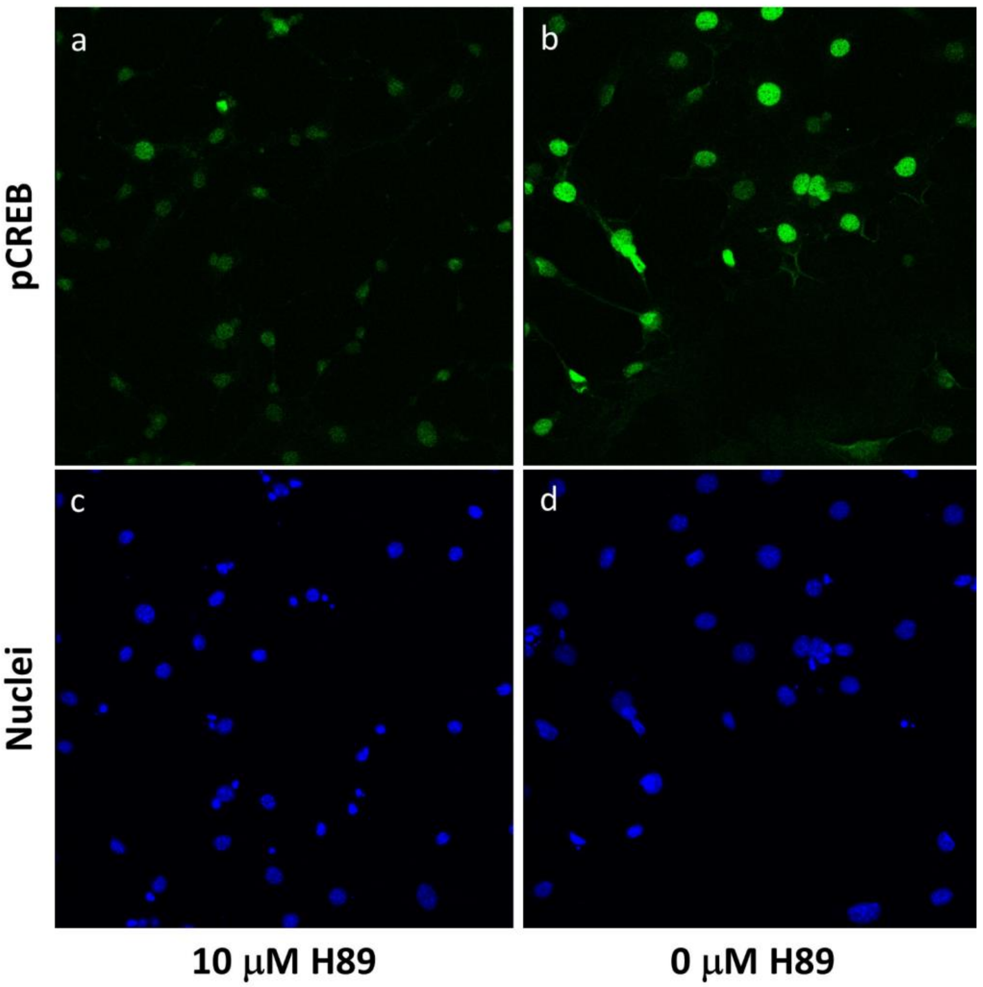

Figure S4 H89 inhibits phosphorylation of CREB at Ser 133. ST*Hdh*^{Q111} cells were treated either 10 μM H89 (a and c) in DMSO or DMSO alone (b and d) under the standard serum-free stress conditions at 37°C for 24 before being fixed and labeled with anti-pCREB (Ser133). The nuclei were stained with Hoechst 33342. Images were acquired with a 40x objective. The pCREB images were scaled to 248 – 7903 gray levels and the nuclei were scaled to 562 – 14649 gray levels. The dimmer intensity of the pCREB in the presence of H89 indicates inhibition of PKA.

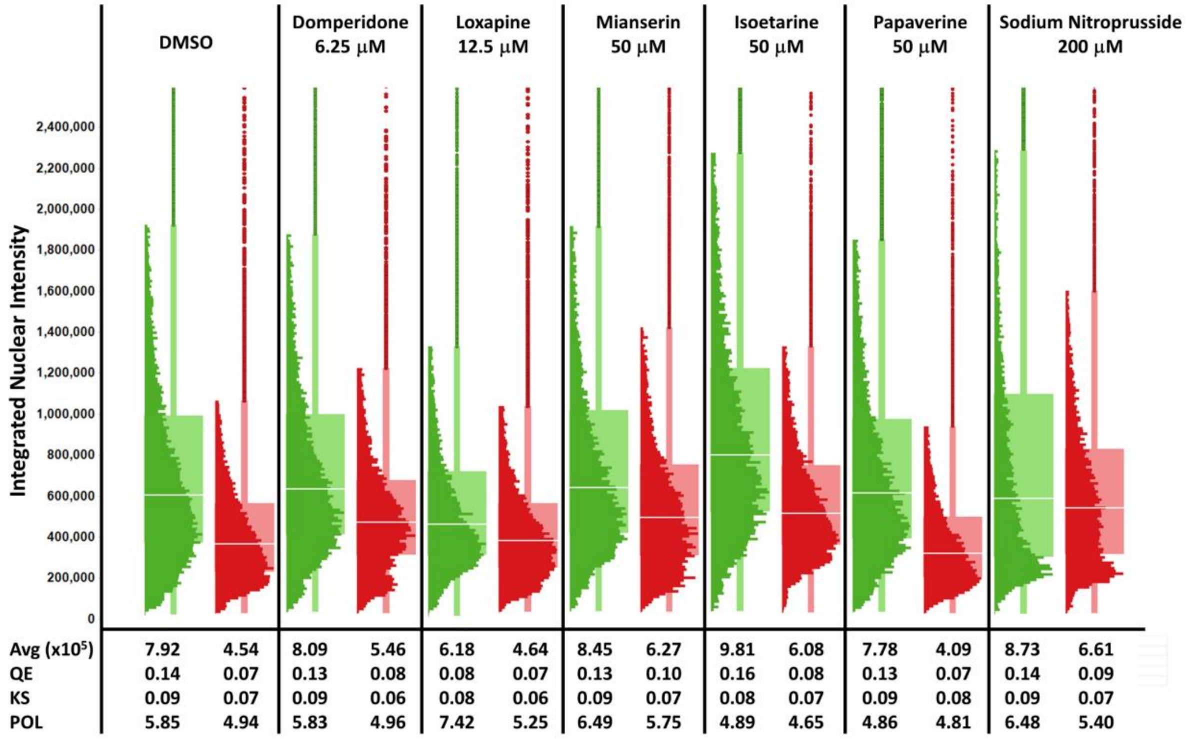

	DMSO		Domperidone 6.25 μM		Loxapine 12.5 μM		Mianserin 50 μM		Isoetarine 50 μM		Papaverine 50 μM		Sodium Nitroprusside 200 μM	
Avg ($x10^5$)	7.92	4.54	8.09	5.46	6.18	4.64	8.45	6.27	9.81	6.08	7.78	4.09	8.73	6.61
QE	0.14	0.07	0.13	0.08	0.08	0.07	0.13	0.10	0.16	0.08	0.13	0.07	0.14	0.09
KS	0.09	0.07	0.09	0.06	0.08	0.06	0.09	0.07	0.08	0.07	0.09	0.08	0.09	0.07
POL	5.85	4.94	5.83	4.96	7.42	5.25	6.49	5.75	4.89	4.65	4.86	4.81	6.48	5.40

Figure S5 PKA activation is heterogeneous within the ST*Hdh*^{Q111} cell population. ST*Hdh*^{Q111} cells were treated with DMSO or protective compounds in the absence (green) or presence (red) of the PKA inhibitor H89 (10 μM) under the standard serum-free stress conditions at 37°C for 24 before being fixed and labeled with anti-pCREB (Ser133). Images were acquired at 10x magnification and analyzed as described in Methods. HistoBox plots[14] were generated showing the distribution of nuclear pCREB levels in the cell population. The plots represent the cumulative data from triplicate replicates run in each of four experiments. The average levels in the populations were calculated along with the PHIs as described by Gough et al.[14]. Quadratic entropy (QE, diversity measure) values > 0.03, Kolmogorov-Smirnov (KS, non-normality measure) values > 0.05, and percent outliers (POL) > 4.5 indicate non-normal, heterogeneous populations. These data show a high degree of heterogeneity in PKA activity as measured by its substrate CREB. Addition of H89 to the cells lowered the degree of heterogeneity in all cases, but did not completely normalize the populations.

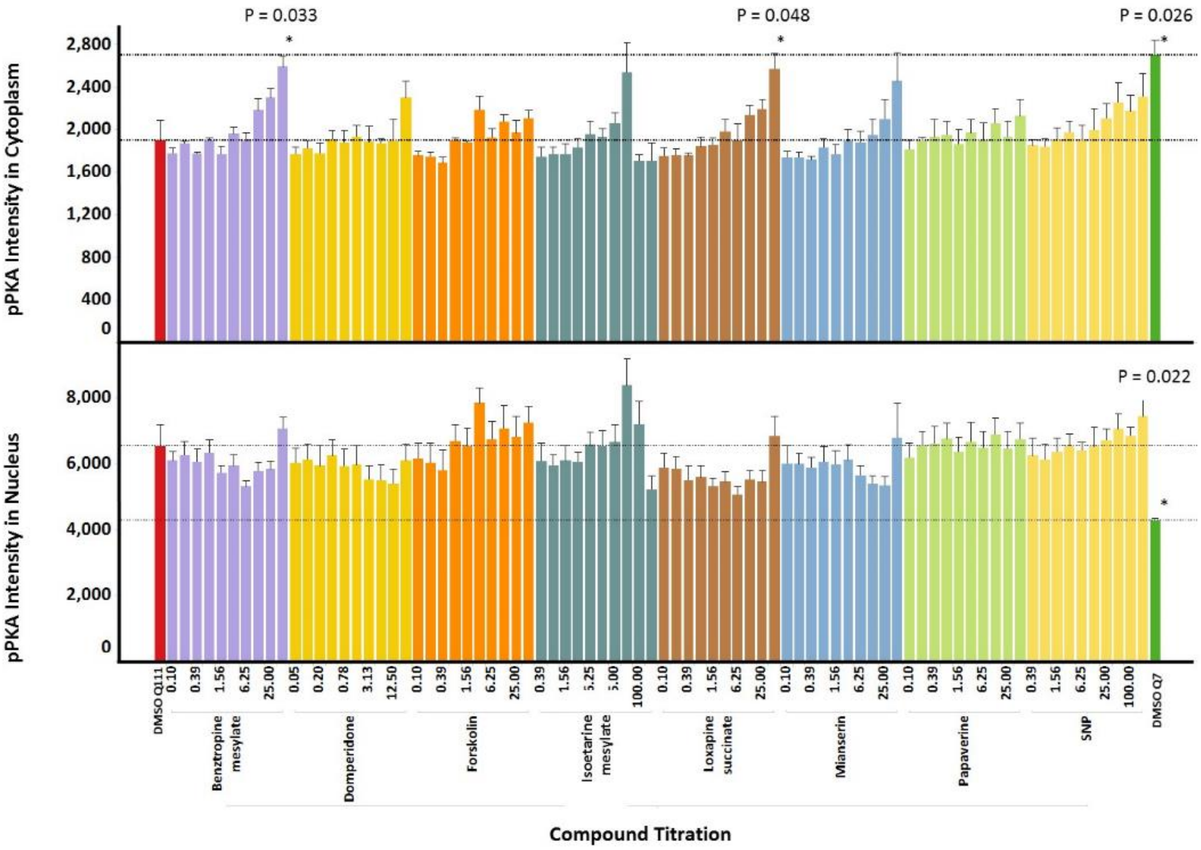

Figure S6. Protective compounds can activate PKA. Cytoplasmic and nuclear pPKA levels were measured in ST*Hdh*^{Q111} cells after incubation with benztropine, domperidone, isoetarine, loxapine, mianserin, papaverine, and Sodium Nitroprusside for 24 h under serum free conditions following the protocol used for the PI protection assay. For cytoplasm levels the upper and lower dotted lines are the average level of ST*Hdh*^{Q7} and ST*Hdh*^{Q111} cells, respectively. For nuclear levels the upper and lower dotted lines are the average level of ST*Hdh*^{111} and ST*Hdh*^{Q7} cells, respectively. Data are the average from three independent experiments (+/- S.E.). T-test was used to assess changes in pPKA levels relative to the ST*Hdh*^{Q111} cells treated with DMSO. SNP = Sodium Nitroprusside.

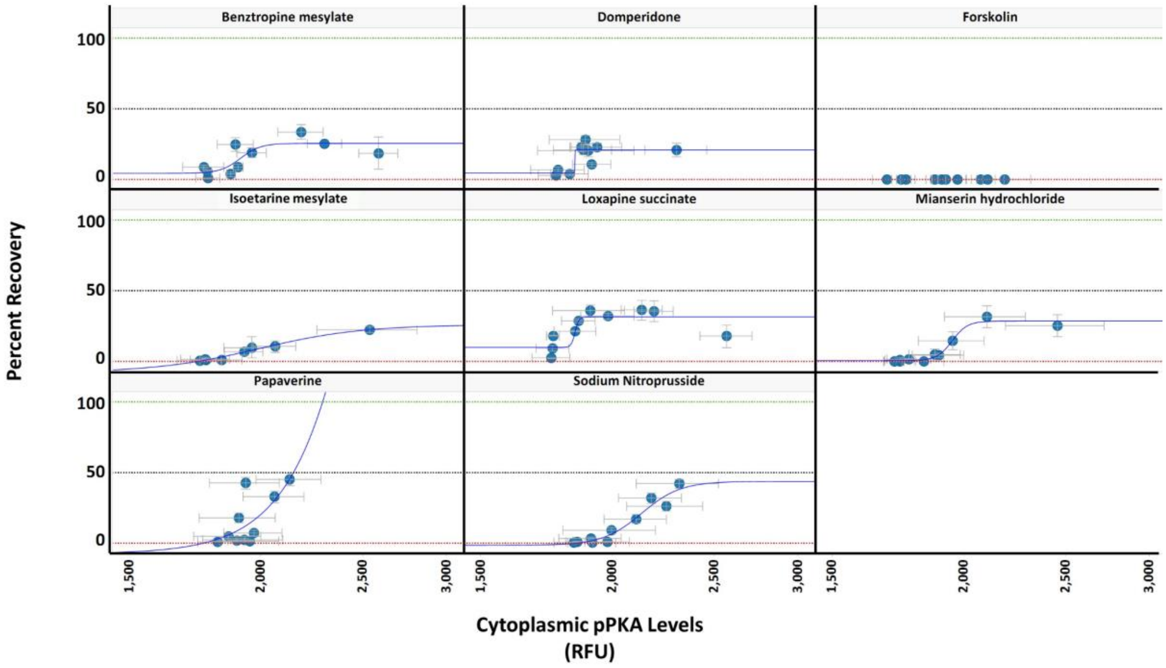

Figure S7. Correlation of Percent Recovery from mHTT toxicity and pPKA levels. The Percent Recovery assessed in the PI assay is plotted against the relative levels of pPKA induced by the compounds measured in the High content assay. All compounds increased pPKA though some were more effective and showed a robust concentration response (see Figure 7 in main text). Different response curves were observed among the protective compounds. Forskolin was not protective, but did show pPKA levels in the range where protection was seen for the other compounds. The Percent Recovery analysis is from triplicate samples run in least two independent runs, and the pPKA analysis is from triplicate samples run in three independent runs (+/- S.E.).

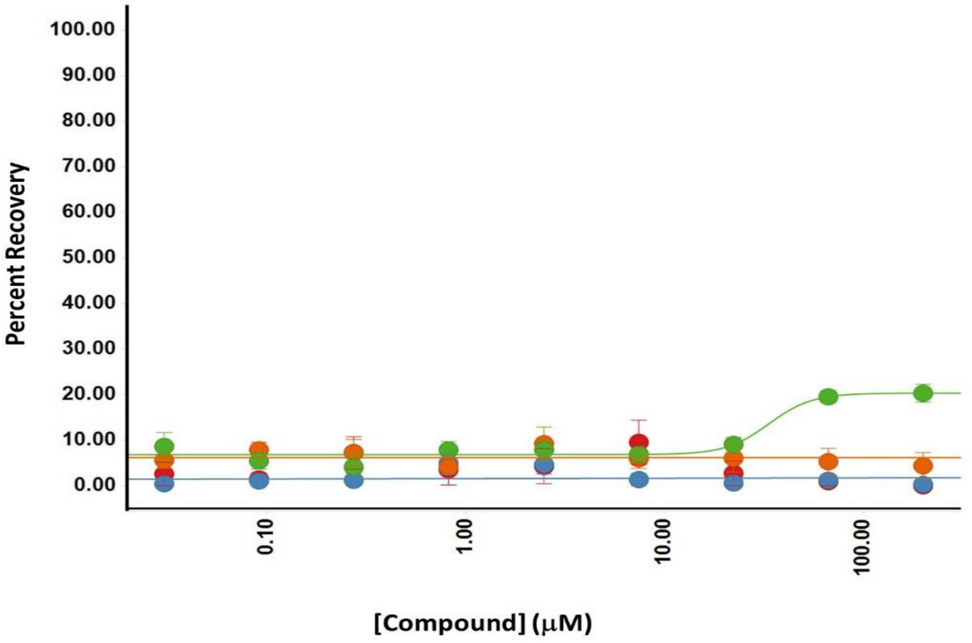

Figure S8. Comparison of carbonic anhydrase inhibitors ethoxzolamide (green), acetazolamide (red), dorzolamide (orange), and brinzolamide (blue) in protecting ST*Hdh*^{Q111} cells. Only ethoxzolamide demonstrated increased protection.

Table S1: Relative solubility of protective compounds

Compound Name	Max Absorbance Wavelength (nM)	Relative Solubility (μM)	n
(Z)-Gugglesterone	250	> 50	2
3-tropanyl-indole-3-carboxylate hydrochloride	230	> 50	3
Beclomethasone	240	> 50	2
Benztropine mesylate	230	> 50	3
Betamethasone	240	> 50	2
Budesonide	250	25	2
Cyproheptadine hydrochloride	230	> 50	2
Domperidone	230	> 50	2
Ethoxzolamide	300	100 - 200	2
Flutamide	230	> 50	2
Hydrocortisone	250	> 50	2
Isoetarine mesylate	230	> 200	2
JWH-015	-	ND	
Lansoprazole	280	> 200	2
Lonidamine	230	> 200	2
Loxapine succinate	230	> 50	3
Meclizine	230	25	2
Mianserin hydrochloride	230	> 50	3
m-Iodobenzylguanidine hemisulfate	230	> 50	2
Papaverine hydrochloride	240	> 50	4
PD 168,077 maleate	230	> 50	3
Quipazine, N-methyl-,dimaleate	240	25	3
Ruthenium red	540	> 50	3
SB 203186	230	> 50	2
Sodium Nitroprusside	230	> 200	2
Tetradecylthioacetic acid	230	> 50	2
Triamcinolone	240	> 50	3
Triprolidine hydrochloride	230	> 50	3
U-83836 dihydrochloride	230	25	2
Vinpocetine	230	> 50	2

Compounds were prepared in DMSO and diluted as describe in Methods. To demonstrate that the maximum concentrations used in the PI assay were below their aqueous solubility limit, 2 μl of the top four DMSO concentrations were diluted in 38 μl PBS (pH 7.4) and their optical absorbance was measured from 230 to 1000 nm. A linear plot of concentration vs peak absorbance (after subtraction of DMSO/PBS blank) indicated that the aqueous solubility limit had not been reach in that concentration range. A plateau in the curve suggested that the solubility limit was being reached for the compound.

Table S2 Targets from DrugBank and STITCH for 32 identified probes

Target ID	Uniprot ID	Target name	Probes Count	Probes
T1	P35367	Histamine H1 receptor	7	Meclizine, Domperidone, Benzatropine, Loxapine, Cyproheptadine, Mianserin, Triprolidine
T2	P28223	5-hydroxytryptamine receptor 2A	6	Loxapine, Benzatropine, Domperidone, Quipazine, N-methyl-,dimaleate, Mianserin, Cyproheptadine
T3	P04150	Glucocorticoid receptor	6	Triamcinolone, Budesonide, Betamethasone, Hydrocortisone, Beclomethasone, Prednisolone
T4	P18825	Alpha-2C adrenergic receptor	5	Mianserin, Benzatropine, Loxapine, Domperidone, Cyproheptadine
T5	P35462	D(3) dopamine receptor	5	Domperidone, Cyproheptadine, Benzatropine, Loxapine, Mianserin
T6	P14416	D(2) dopamine receptor	5	Benzatropine, Mianserin, Domperidone, Loxapine, Cyproheptadine
T7	P28335	5-hydroxytryptamine receptor 2C	5	Mianserin, Loxapine, Benzatropine, Quipazine, N-methyl-,dimaleate, Cyproheptadine
T8	P08172	Muscarinic acetylcholine receptor M2	4	Benzatropine, Loxapine, Cyproheptadine, Mianserin
T9	P08173	Muscarinic acetylcholine receptor M4	4	Loxapine, Cyproheptadine, Benzatropine, Mianserin
T10	P11229	Muscarinic acetylcholine receptor M1	4	Mianserin, Loxapine, Cyproheptadine, Benzatropine
T11	P08913	Alpha-2A adrenergic receptor	4	Benzatropine, Mianserin, Cyproheptadine, Loxapine
T12	P18089	Alpha-2B adrenergic receptor	4	Benzatropine, Cyproheptadine, Loxapine, Mianserin
T13	P41595	5-hydroxytryptamine receptor 2B	4	Benzatropine, Quipazine, N-methyl-,dimaleate, Mianserin, Cyproheptadine
T14	P08912	Muscarinic acetylcholine receptor M5	4	Cyproheptadine, Loxapine, Mianserin, Benzatropine
T15	P20309	Muscarinic acetylcholine receptor M3	4	Cyproheptadine, Benzatropine, Loxapine, Mianserin
T16	P34969	5-hydroxytryptamine receptor 7	3	Loxapine, Cyproheptadine, Mianserin
T17	Q9H3N8	Histamine H4 receptor	3	Cyproheptadine, Mianserin, Loxapine
T18	Q01959	Sodium-dependent dopamine transporter	3	Loxapine, Benzatropine, Mianserin
T19	P21728	D(1A) dopamine receptor	3	Cyproheptadine, Loxapine, Mianserin
T20	P25100	Alpha-1D adrenergic receptor	3	Cyproheptadine, Mianserin, Benzatropine
T21	P50406	5-hydroxytryptamine receptor 6	3	Cyproheptadine, Mianserin, Loxapine
T22	P08908	5-hydroxytryptamine receptor 1A	3	Mianserin, Loxapine, Cyproheptadine
T23	P31645	Sodium-dependent serotonin transporter	3	Quipazine, N-methyl-,dimaleate, Loxapine, Mianserin
T24	P28221	5-hydroxytryptamine receptor 1D	2	Mianserin, Loxapine
T25	P23975	Sodium-dependent noradrenaline transporter	2	Loxapine, Mianserin
T26	P35368	Alpha-1B adrenergic receptor	2	Loxapine, Mianserin
T27	P21918	D(1B) dopamine receptor	2	Loxapine, Mianserin
T28	P35348	Alpha-1A adrenergic receptor	2	Loxapine, Mianserin
T29	P25021	Histamine H2 receptor	2	Cyproheptadine, Loxapine
T30	P08588	Beta-1 adrenergic receptor	2	Loxapine, Isoetarine
T31	P98153	Integral membrane protein DGCR2/IDD	1	Ethoxzolamide
T32	Q9UBN7	Histone deacetylase 6	1	Vorinostat
T33	Q9ULX7	Carbonic anhydrase 14	1	Ethoxzolamide
T34	P04083	Annexin A1	1	Hydrocortisone
T35	P10636	Microtubule-associated protein tau	1	Lansoprazole
T36	P41145	Kappa-type opioid receptor	1	Mianserin
T37	Q9UKV0	Histone deacetylase 9	1	Vorinostat
T38	P47898	5-hydroxytryptamine receptor 5A	1	Loxapine
T39	Q16790	Carbonic anhydrase 9	1	Ethoxzolamide
T40	P35218	Carbonic anhydrase 5A, mitochondrial	1	Ethoxzolamide
T41	Q8N1Q1	Carbonic anhydrase 13	1	Ethoxzolamide
T42	Q13547	Histone deacetylase 1	1	Vorinostat
T43	P46098	5-hydroxytryptamine receptor 3A	1	Loxapine
T44	P28566	5-hydroxytryptamine receptor 1E	1	Loxapine
T45	Q9BY41	Histone deacetylase 8	1	Vorinostat
T46	Q99720	Sigma non-opioid intracellular receptor 1	1	Benzatropine
T47	Q12809	Potassium voltage-gated channel subfamily H member 2	1	Domperidone
T48	P30939	5-hydroxytryptamine receptor 1F	1	Mianserin
T49	P35869	Aryl hydrocarbon receptor	1	Flutamide
T50	P43166	Carbonic anhydrase 7	1	Ethoxzolamide
T51	Q14432	cGMP-inhibited 3',5'-cyclic phosphodiesterase A	1	Papaverine
T52	P28222	5-hydroxytryptamine receptor 1B	1	Loxapine
T53	Q969S8	Histone deacetylase 10	1	Vorinostat
T54	P16066	Atrial natriuretic peptide receptor 1	1	Nitroprusside
T55	P23280	Carbonic anhydrase 6 (EC 4.2.1.1)	1	Ethoxzolamide
T56	Q8WUI4	Histone deacetylase 7	1	Vorinostat
T57	P56524	Histone deacetylase 4	1	Vorinostat
T58	Q96DB2	Histone deacetylase 11	1	Vorinostat
T59	P08185	Corticosteroid-binding globulin	1	Hydrocortisone
T60	O43570	Carbonic anhydrase 12	1	Ethoxzolamide
T61	P07550	Beta-2 adrenergic receptor	1	Isoetarine
T62	P21917	D(4) dopamine receptor	1	Loxapine
T63	Q9Y2D0	Carbonic anhydrase 5B, mitochondrial	1	Ethoxzolamide
T64	P00918	Carbonic anhydrase 2	1	Ethoxzolamide
T65	P22748	Carbonic anhydrase 4	1	Ethoxzolamide
T66	P34972	Cannabinoid receptor 2	1	JWH-015
T67	Q9UQL6	Histone deacetylase 5	1	Vorinostat
T68	P20648	Potassium-transporting ATPase alpha chain 1	1	Lansoprazole
T69	P10275	Androgen receptor	1	Flutamide
T70	Q9Y233	cAMP and cAMP-inhibited cGMP 3',5'-cyclic phosphodiesterase 10A	1	Papaverine
T71	P00915	Carbonic anhydrase 1	1	Ethoxzolamide
T72	P21554	Cannabinoid receptor 1	1	JWH-015
T73	Q92769	Histone deacetylase 2	1	Vorinostat
T74	Q07343	cAMP-specific 3',5'-cyclic phosphodiesterase 4B	1	Papaverine
T75	O15379	Histone deacetylase 3	1	Vorinostat

Note:
Targets were ranked by the number of intersecting probes, probes interact with each target were listed in the corresponding row.

Table S3 Mapping of 32 identified probes and targets in KEGG human pathways

Index	Pathway Name	Probes count	Targets in this pathway	Probes in this pathway
1	Calcium signaling pathway	9	T8, T2, T10, T27, T7, T29, T19, T13, T16, T21, T14, T30, T20, T15, T26, T1, T61, T38, T28	Mianserin, Domperidone, Benzatropine, Cyproheptadine, (Quipazine,N-methyl-,dimaleate), Loxapine, Isoetarine, Meclizine, Triprolidine
2	Inflammatory mediator regulation of TRP channels	8	T1, T7, T2, T13	Mianserin, Domperidone, Benzatropine, Cyproheptadine, (Quipazine,N-methyl-,dimaleate), Loxapine, Meclizine, Triprolidine
3	cGMP-PKG signaling pathway	8	T54, T4, T51, T20, T30, T11, T12, T26, T61, T28	Nitroprusside, Mianserin, Domperidone, Benzatropine, Cyproheptadine, Loxapine, Papaverine, Isoetarine
4	cAMP signaling pathway	8	T8, T54, T10, T27, T44, T52, T51, T24, T48, T21, T19, T30, T6, T22, T74, T61	Nitroprusside, Mianserin, Domperidone, Benzatropine, Cyproheptadine, Loxapine, Papaverine, Isoetarine
5	Gap junction	7	T2, T7, T13, T19, T30, T6	Mianserin, Domperidone, Benzatropine, Cyproheptadine, (Quipazine,N-methyl-,dimaleate), Loxapine, Isoetarine
6	Alcoholism	6	T32, T56, T75, T58, T18, T53, T42, T73, T45, T6, T67, T37, T19, T57	Vorinostat, Mianserin, Domperidone, Benzatropine, Cyproheptadine, Loxapine
7	Rap1 signaling pathway	6	T72, T6	Mianserin, Domperidone, Benzatropine, JWH-015, Cyproheptadine, Loxapine
8	Serotonergic synapse	6	T43, T44, T2, T24, T52, T7, T13, T48, T21, T16, T22, T38, T23	Mianserin, Domperidone, Benzatropine, Cyproheptadine, Quipazine, N-methyl-,dimaleate, Loxapine
9	Amphetamine addiction	5	T19, T18, T42	Loxapine, Benzatropine, Vorinostat, Cyproheptadine, Mianserin
10	Dopaminergic synapse	5	T5, T18, T27, T19, T6, T62	Loxapine, Domperidone, Benzatropine, Cyproheptadine, Mianserin
11	Cocaine addiction	5	T19, T18, T6	Loxapine, Domperidone, Benzatropine, Cyproheptadine, Mianserin
12	Parkinson's disease	5	T19, T18, T6	Loxapine, Domperidone, Benzatropine, Cyproheptadine, Mianserin
13	Morphine addiction	4	T74, T19, T51, T70	Loxapine, Papaverine, Mianserin, Cyproheptadine
14	Cholinergic synapse	4	T8, T9, T14, T10, T15	Loxapine, Benzatropine, Cyproheptadine, Mianserin
15	PI3K-Akt signaling pathway	4	T8, T10	Loxapine, Benzatropine, Cyproheptadine, Mianserin
16	Ras signaling pathway	3	T16	Loxapine, Mianserin, Cyproheptadine
17	Purine metabolism	2	T74, T54, T51, T70	Papaverine, Nitroprusside
18	Endocytosis	2	T61, T30	Loxapine, Isoetarine
19	AMPK signaling pathway	2	T28	Loxapine, Mianserin
20	Pathways in cancer	2	T69, T73, T42	Flutamide, Vorinostat
21	Oxidative phosphorylation	1	T68	Lansoprazole
22	Retrograde endocannabinoid signaling	1	T72	JWH-015
23	Alzheimer's disease	1	T35	Lansoprazole
24	Proximal tubule bicarbonate reclamation	1	T64, T65	Ethoxzolamide
25	Huntington's disease	1	T73, T42	Vorinostat
26	Nitrogen metabolism	1	T64, T65, T63, T50, T55, T33, T71, T39, T60, T40, T41	Ethoxzolamide
27	Cell cycle	1	T73, T42	Vorinostat
28	Transcriptional misregulation in cancer	1	T73, T42	Vorinostat
29	Longevity regulating pathway - multiple species	1	T73, T42	Vorinostat
30	MicroRNAs in cancer	1	T57, T42	Vorinostat
31	Notch signaling pathway	1	T73, T42	Vorinostat
32	MAPK signaling pathway	1	T35	Lansoprazole
33	Epstein-Barr virus infection	1	T57, T67, T73, T42	Vorinostat
34	Viral carcinogenesis	1	T32, T58, T75, T53, T42, T73, T45, T56, T67, T37, T57	Vorinostat

Note:
Pathways were ranked by the number of mapped probes, probe targets that mapped into each pathway and the corresponding probes were listed in the corresponding pathway row. Target information for each target ID is listed in Supplementary Table 2.

Table S4 - Combination Pairs

Combination Number	Combination	Combination Number	Combination
1	Betamethasone_Lonidamine	81	Ruthenium red_Budesonide
2	Sodium Nitroprusside_Triamcinolone	82	Ruthenium red_3-tropanyl-indole-3-carboxylate hydrochloride
3	Sodium Nitroprusside_Betamethasone	83	Triprolidine hydrochloride_3-tropanyl-indole-3-carboxylate hydrochloride
4	Sodium Nitroprusside_Beclomethasone	84	Beclomethasone_Budesonide
5	Ethoxzolamide_Beclomethasone	85	Ethoxzolamide_JWH-015
6	Triprolidine hydrochloride_Betamethasone	86	Triprolidine hydrochloride_Domperidone
7	Domperidone_Isoetarine mesylate	87	Triprolidine hydrochloride_Quipazine,N-methyl-,dimaleate
8	Sodium Nitroprusside_Budesonide	88	Triamcinolone_3-tropanyl-indole-3-carboxylate hydrochloride
9	Isoetarine mesylate_m-Iodobenzylguanidine hemisulfate	89	Ethoxzolamide_Lansoprazole
10	Sodium Nitroprusside_Isoetarine mesylate	90	Beclomethasone_Betamethasone
11	Sodium Nitroprusside_Lansoprazole	91	Ethoxzolamide_Mianserin hydrochloride
12	Ethoxzolamide_Betamethasone	92	Ethoxzolamide_m-Iodobenzylguanidine hemisulfate
13	Sodium Nitroprusside_Mianserin hydrochloride	93	Budesonide_3-tropanyl-indole-3-carboxylate hydrochloride
14	Beclomethasone_Quipazine,N-methyl-,dimaleate	94	Ruthenium red_Lonidamine
15	Sodium Nitroprusside_Loxapine succinate	95	Triprolidine hydrochloride_Budesonide
16	Ethoxzolamide_Loxapine succinate	96	Triamcinolone_Cyproheptadine hydrochloride
17	Ethoxzolamide_Domperidone	97	3-tropanyl-indole-3-carboxylate hydrochloride_PD168,077 maleate
18	Ruthenium red_Betamethasone	98	Ethoxzolamide_PD168,077 maleate
19	3-tropanyl-indole-3-carboxylate hydrochloride_Isoetarine mesylate	99	Budesonide_Isoetarine mesylate
20	Benztropine mesylate_Isoetarine mesylate	100	Triamcinolone_Quipazine,N-methyl-,dimaleate
21	Isoetarine mesylate_Loxapine succinate	101	Ruthenium red_Benztropine mesylate
22	Domperidone_m-Iodobenzylguanidine hemisulfate	102	Triamcinolone_Budesonide
23	Sodium Nitroprusside_U-83836 dihydrochloride	103	Ruthenium red_Triprolidine hydrochloride
24	Tetradecylthioacetic acid_Budesonide	104	Sodium Nitroprusside_Cyproheptadine hydrochloride
25	Betamethasone_Quipazine,N-methyl-,dimaleate	105	3-tropanyl-indole-3-carboxylate hydrochloride_Papaverine hydrochloride
26	Tetradecylthioacetic acid_Betamethasone	106	Ethoxzolamide_Isoetarine mesylate
27	Tetradecylthioacetic acid_Isoetarine mesylate	107	Lonidamine_Benztropine mesylate
28	Isoetarine mesylate_Mianserin hydrochloride	108	3-tropanyl-indole-3-carboxylate hydrochloride_Mianserin hydrochloride
29	Isoetarine mesylate_Papaverine hydrochloride	109	Sodium Nitroprusside_Ethoxzolamide
30	Betamethasone_Isoetarine mesylate	110	Lansoprazole_Loxapine succinate
31	Triamcinolone_Benztropine mesylate	111	Ethoxzolamide_Papaverine hydrochloride
32	Domperidone_Lansoprazole	112	Ruthenium red_Quipazine,N-methyl-,dimaleate
33	Beclomethasone_Isoetarine mesylate	113	Mianserin hydrochloride_Papaverine hydrochloride
34	Sodium Nitroprusside_Lonidamine	114	Tetradecylthioacetic acid_Flutamide
35	Triprolidine hydrochloride_Beclomethasone	115	Mianserin hydrochloride_PD168,077 maleate
36	Triamcinolone_Lonidamine	116	Domperidone_Loxapine succinate
37	Beclomethasone_3-tropanyl-indole-3-carboxylate hydrochloride	117	Lonidamine_Domperidone
38	Betamethasone_3-tropanyl-indole-3-carboxylate hydrochloride	118	Benztropine mesylate_m-Iodobenzylguanidine hemisulfate
39	Beclomethasone_Domperidone	119	Flutamide_Loxapine succinate
40	Tetradecylthioacetic acid_Triamcinolone	120	Tetradecylthioacetic acid_Quipazine,N-methyl-,dimaleate
41	Sodium Nitroprusside_Triprolidine hydrochloride	121	Ruthenium red_Isoetarine mesylate
42	Triamcinolone_Domperidone	122	Tetradecylthioacetic acid_3-tropanyl-indole-3-carboxylate hydrochloride
43	Ethoxzolamide_Budesonide	123	Tetradecylthioacetic acid_Lonidamine
44	Domperidone_Papaverine hydrochloride	124	Domperidone_Mianserin hydrochloride
45	Isoetarine mesylate_PD168,077 maleate	125	Triprolidine hydrochloride_Benztropine mesylate
46	Ethoxzolamide_Triamcinolone	126	Ethoxzolamide_Ruthenium red
47	Sodium Nitroprusside_3-tropanyl-indole-3-carboxylate hydrochloride	127	Lansoprazole_m-Iodobenzylguanidine hemisulfate
48	Betamethasone_Benztropine mesylate	128	Loxapine succinate_Mianserin hydrochloride
49	Ethoxzolamide_Lonidamine	129	Benztropine mesylate_Papaverine hydrochloride
50	Triamcinolone_Isoetarine mesylate	130	Ruthenium red_Triamcinolone
51	Domperidone_PD168,077 maleate	131	Triamcinolone_Beclomethasone
52	Ethoxzolamide_Triprolidine hydrochloride	132	Domperidone_Flutamide
53	Loxapine succinate_m-Iodobenzylguanidine hemisulfate	133	3-tropanyl-indole-3-carboxylate hydrochloride_Loxapine succinate
54	Budesonide_Quipazine,N-methyl-,dimaleate	134	Lonidamine_Quipazine,N-methyl-,dimaleate
55	Beclomethasone_Lonidamine	135	Tetradecylthioacetic acid_Benztropine mesylate
56	Sodium Nitroprusside_Domperidone	136	Cyproheptadine hydrochloride_Lonidamine
57	Ethoxzolamide_Benztropine mesylate	137	Tetradecylthioacetic acid_Triprolidine hydrochloride
58	Ruthenium red_Domperidone	138	Tetradecylthioacetic acid_Domperidone
59	Ethoxzolamide_Quipazine,N-methyl-,dimaleate	139	Budesonide_Benztropine mesylate
60	Loxapine succinate_PD168,077 maleate	140	Cyproheptadine hydrochloride_Quipazine,N-methyl-,dimaleate
61	3-tropanyl-indole-3-carboxylate hydrochloride_m-Iodobenzylguanidine hemisu	141	Beclomethasone_Cyproheptadine hydrochloride
62	Budesonide_Lonidamine	142	Benztropine mesylate_Loxapine succinate
63	Triamcinolone_Triprolidine hydrochloride	143	Papaverine hydrochloride_PD168,077 maleate
64	Sodium Nitroprusside_Benztropine mesylate	144	Betamethasone_Cyproheptadine hydrochloride
65	Triprolidine hydrochloride_Isoetarine mesylate	145	Triprolidine hydrochloride_Flutamide
66	Triprolidine hydrochloride_Lonidamine	146	m-Iodobenzylguanidine hemisulfate_PD168,077 maleate
67	Tetradecylthioacetic acid_(Z)-Gugglesterone	147	Lonidamine_3-tropanyl-indole-3-carboxylate hydrochloride
68	Ethoxzolamide_3-tropanyl-indole-3-carboxylate hydrochloride	148	Ethoxzolamide_Cyproheptadine hydrochloride
69	Beclomethasone_Benztropine mesylate	149	Tetradecylthioacetic acid_Cyproheptadine hydrochloride
70	Loxapine succinate_Papaverine hydrochloride	150	m-Iodobenzylguanidine hemisulfate_Papaverine hydrochloride
71	Ruthenium red_Beclomethasone	151	Ruthenium red_Cyproheptadine hydrochloride
72	Ethoxzolamide_Tetradecylthioacetic acid	152	Benztropine mesylate_Domperidone
73	Lonidamine_Isoetarine mesylate	153	Sodium Nitroprusside_Tetradecylthioacetic acid
74	Triamcinolone_Betamethasone	154	Triprolidine hydrochloride_Cyproheptadine hydrochloride
75	Betamethasone_Budesonide	155	Sodium Nitroprusside_Ruthenium red
76	Lonidamine_Flutamide	156	Isoetarine mesylate_Lansoprazole
77	Triamcinolone_Flutamide	157	Flutamide_m-Iodobenzylguanidine hemisulfate
78	Budesonide_Domperidone	158	3-tropanyl-indole-3-carboxylate hydrochloride_Benztropine mesylate
79	Tetradecylthioacetic acid_Beclomethasone	159	Ruthenium red_Tetradecylthioacetic acid
80	Ethoxzolamide_Flutamide		

Table S5 - Synergistic compounds

Combination Number	Combination	Avg Percent Recovery	Std	n*	Avg Combi Ratio	Std	Median BCI
1	Betamethasone_Lonidamine	84.68	6.12	4	1.98	0.62	1.39
2	Sodium Nitroprusside_Triamcinolone	89.08	4.62	5	1.89	0.34	1.34
3	Sodium Nitroprusside_Betamethasone	96.89	4.67	5	1.81	0.39	1.27
4	Sodium Nitroprusside_Beclomethasone	94.51	2.60	5	1.78	0.26	1.26
5	Ethoxzolamide_Beclomethasone	86.85	2.49	5	1.72	0.11	1.23
6	Triprolidine hydrochloride_Betamethasone	91.41	2.80	4	1.59	0.28	1.22
7	Domperidone_Isoetarine mesylate	78.69	12.41	4	1.79	0.12	1.20
8	Sodium Nitroprusside_Budesonide	100.88	1.04	5	1.50	0.08	1.19
9	Isoetarine mesylate_m-Iodobenzylguanidine hemisulfate	71.92	6.76	4	1.86	0.06	1.17
10	Sodium Nitroprusside_Isoetarine mesylate	80.63	5.73	5	1.87	0.36	1.17
11	Sodium Nitroprusside_Lansoprazole	82.22	6.02	4	1.56	0.08	1.17
12	Ethoxzolamide_Betamethasone	77.30	33.72	6	1.55	0.02	1.15
13	Sodium Nitroprusside_Mianserin hydrochloride	75.42	8.10	4	1.63	0.20	1.14
14	Beclomethasone_Quipazine,N-methyl-,dimaleate	79.64	2.89	2	1.66	0.15	1.14
15	Sodium Nitroprusside_Loxapine succinate	80.46	2.19	4	1.49	0.03	1.13
16	Ethoxzolamide_Loxapine succinate	74.43	4.13	4	1.38	0.03	1.13
17	Ethoxzolamide_Domperidone	81.30	17.04	6	1.68	0.27	1.12
18	Ruthenium red_Betamethasone	79.17	7.01	4	1.51	0.34	1.12
19	3-tropanyl-indole-3-carboxylate hydrochloride_Isoetarine mesylate	69.62	3.52	4	1.76	0.08	1.12
20	Benztropine mesylate_Isoetarine mesylate	65.81	2.06	4	1.71	0.24	1.12
21	Isoetarine mesylate_Loxapine succinate	80.01	4.50	4	1.48	0.06	1.11
22	Domperidone_m-Iodobenzylguanidine hemisulfate	72.00	3.99	4	1.64	0.04	1.11
23	Sodium Nitroprusside_U-83836 dihydrochloride	84.34	2.71	5	1.45	0.12	1.11
24	Tetradecylthioacetic acid_Budesonide	96.68	1.02	4	1.43	0.07	1.11
25	Betamethasone_Quipazine,N-methyl-,dimaleate	83.07	0.57	2	1.50	0.12	1.11
26	Tetradecylthioacetic acid_Betamethasone	85.04	3.50	4	1.42	0.19	1.11
27	Tetradecylthioacetic acid_Isoetarine mesylate	88.66	4.19	2	1.39	0.08	1.10
28	Isoetarine mesylate_Mianserin hydrochloride	73.47	5.54	4	1.60	0.34	1.10
29	Isoetarine mesylate_Papaverine hydrochloride	74.95	6.28	4	1.57	0.02	1.10
30	Betamethasone_Isoetarine mesylate	82.64	9.58	2	1.48	0.04	1.10
31	Triamcinolone_Benztropine mesylate	74.81	0.50	2	1.31	0.30	1.09
32	Domperidone_Lansoprazole	79.79	12.84	4	1.51	0.11	1.08
33	Beclomethasone_Isoetarine mesylate	77.04	5.53	2	1.47	0.01	1.08
34	Sodium Nitroprusside_Lonidamine	66.90	22.35	5	1.39	0.09	1.08
35	Triprolidine hydrochloride_Beclomethasone	86.17	3.73	4	1.42	0.15	1.08
36	Triamcinolone_Lonidamine	58.55	8.25	4	1.72	0.49	1.07
37	Beclomethasone_3-tropanyl-indole-3-carboxylate hydrochloride	78.29	3.15	2	1.53	0.01	1.07
38	Betamethasone_3-tropanyl-indole-3-carboxylate hydrochloride	82.88	8.42	2	1.49	0.02	1.07
39	Beclomethasone_Domperidone	83.70	0.17	2	1.42	0.02	1.07
40	Tetradecylthioacetic acid_Triamcinolone	77.97	5.99	4	1.32	0.29	1.07
41	Sodium Nitroprusside_Triprolidine hydrochloride	86.41	5.97	5	1.46	0.25	1.07
42	Triamcinolone_Domperidone	73.41	1.45	2	1.25	0.01	1.07
43	Ethoxzolamide_Budesonide	87.19	3.45	6	1.29	0.03	1.07
44	Domperidone_Papaverine hydrochloride	75.42	11.88	4	1.58	0.19	1.07
45	Isoetarine mesylate_PD168,077 maleate	71.38	2.48	4	1.57	0.15	1.07
46	Ethoxzolamide_Triamcinolone	61.43	7.36	6	1.54	0.33	1.06
47	Sodium Nitroprusside_3-tropanyl-indole-3-carboxylate hydrochloride	68.86	12.35	5	1.53	0.13	1.06
48	Betamethasone_Benztropine mesylate	85.13	0.92	2	1.44	0.29	1.05
49	Ethoxzolamide_Lonidamine	64.69	6.30	6	1.61	0.37	1.05
50	Triamcinolone_Isoetarine mesylate	61.87	12.97	2	1.39	0.12	1.05
51	Domperidone_PD168,077 maleate	72.46	5.13	4	1.55	0.17	1.04
52	Ethoxzolamide_Triprolidine hydrochloride	77.82	7.22	6	1.29	0.17	1.04
53	Loxapine succinate_m-Iodobenzylguanidine hemisulfate	73.76	2.68	4	1.36	0.00	1.04
54	Budesonide_Quipazine,N-methyl-,dimaleate	85.41	12.39	2	1.25	0.12	1.04
55	Beclomethasone_Lonidamine	69.30	21.92	4	1.37	0.03	1.03
56	Sodium Nitroprusside_Domperidone	61.74	25.78	5	1.26	0.37	1.03
57	Ethoxzolamide_Benztropine mesylate	58.69	10.99	6	1.46	0.23	1.02
58	Ruthenium red_Domperidone	80.82	1.76	2	1.37	0.05	1.01
59	Ethoxzolamide_Quipazine,N-methyl-,dimaleate	60.16	7.97	4	1.59	0.17	1.01
60	Loxapine succinate_PD168,077 maleate	75.92	2.61	4	1.40	0.01	1.01
61	3-tropanyl-indole-3-carboxylate hydrochloride_m-Iodobenzylguanidine hemisulfate	61.79	2.27	4	1.57	0.07	1.01

* Compound combinations were run at least once on two different days. n= the total number of combination samples analyzed.
An n=2 indictes a combination was run only once on each of the two days.

Table S6: Synergistic compound pairs that converge on PKA/PKG signaling.

Combination	Compound 1 MOA	Compound 2 MOA
Sodium Nitroprusside_Loxapine succinate	Nitric oxide synthase	D2/3 receptor antagonist
Sodium Nitroprusside_Domperidone	Nitric oxide synthase	D2/3 receptor antagonist
Sodium Nitroprusside_ Mianserin HCl	Nitric oxide synthase	5HT and a-2C adrenergic antagonist
Isoetarine mesylate_Loxapine succinate	β-1/2 adrenergic receptor agonist	D2/3 receptor antagonist
Isoetarine mesylate_Papaverine HCl	β-1/2 adrenergic receptor agonist	PDE10A inhibitor
Domperidone_Papaverine HCl	D2/3 receptor antagonist	PDE10A inhibitor
Isoetarine mesylate_Mianserin HCl	β-1/2 adrenergic receptor agonist	5HT and a-2C adrenergic antagonist
Sodium Nitroprusside_Isoetarine mesylate	Nitric oxide synthase	β-1/2 adrenergic receptor agonist
Benztropine mesylate_Isoetarine mesylate	M1 receptor antagonist	β-1/2 adrenergic receptor agonist
Domperidone_Isoetarine mesylate	D2/3 receptor antagonist	β-1/2 adrenergic receptor agonist
